# Prolonged administration of total glucosides of paeony improves intestinal immune imbalance and epithelial barrier damage in collagen-induced arthritis rats based on metabolomics-network pharmacology integrated analysis

**DOI:** 10.3389/fphar.2023.1187797

**Published:** 2023-11-13

**Authors:** Rui Xu, Jine Peng, Zhe Ma, Kaili Xie, Meijing Li, Qi Wang, Xiaomeng Guo, Nan Nan, Sihui Wang, Jing Li, Jingjing Xu, Muxin Gong

**Affiliations:** ^1^ School of Traditional Chinese Medicine, Capital Medical University, Beijing, China; ^2^ Beijing Key Laboratory of Traditional Chinese Medicine Collateral Disease Theory Research, Beijing, China; ^3^ Department of Pharmacy, Beijing Ditan Hospital, Capital Medical University, Beijing, China

**Keywords:** rheumatoid arthritis, total glucosides of paeony, collagen-induced arthritis, metabolomics, network pharmacology, intestinal mucosal immunity

## Abstract

Rheumatoid arthritis (RA) is a chronic systemic autoimmune disease characterized by synovial inflammation and joint damage with complex pathological mechanisms. In recent years, many studies have shown that the dysregulation of intestinal mucosal immunity and the damage of the epithelial barrier are closely related to the occurrence of RA. Total glucosides of paeony (TGP) have been used clinically for the treatment of RA in China for decades, while the pharmacological mechanism is still uncertain. The purpose of this study was to investigate the regulatory effect and mechanism of TGP on intestinal immunity and epithelial barrier in RA model rats. The results showed that TGP alleviated immune hyperfunction by regulating the ratio of CD3^+^, CD4^+^ and CD8^+^ in different lymphocyte synthesis sites of the small intestine, including Peyer’s patches (PPs), intraepithelial lymphocytes (IELs), and lamina propria lymphocytes (LPLs). Specially, TGP first exhibited immunomodulatory effects on sites close to the intestinal lumen (IELs and LPLs), and then on PPs far away from the intestinal lumen as the administration time prolonged. Meanwhile, TGP restores the intestinal epithelial barrier by upregulating the ratio of villi height (V)/crypt depth (C) and expression of tight junction proteins (ZO-1, occludin). Finally, the integrated analysis of metabolomics-network pharmacology was also used to explore the possible regulation mechanism of TGP on the intestinal tract. Metabolomics analysis revealed that TGP reversed the intestinal metabolic profile disturbance in CIA rats, and identified 32 biomarkers and 163 corresponding targets; network pharmacology analysis identified 111 potential targets for TGP to treat RA. By intersecting the results of the two, three key targets such as ADA, PNP and TYR were determined. Pharmacological verification experiments showed that the levels of ADA and PNP in the small intestine of CIA rats were significantly increased, while TGP significantly decreased their ADA and PNP levels. In conclusion, purine metabolism may play an important role in the process of TGP improving RA-induced intestinal immune imbalance and impaired epithelial barrier.

## Graphical Abstract

**Figure:**
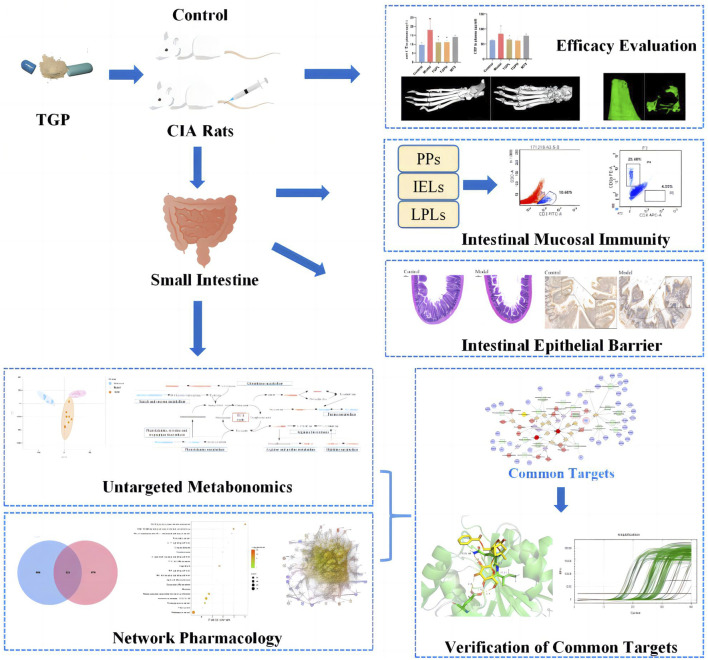
Summary of experiment processes.

## 1 Introduction

Rheumatoid arthritis (RA) is an autoimmune disease characterized by chronic inflammatory lesions of joint tissues, which display pain, swelling, and decreased function of the affected joints in clinical (Khanna et al., 2021). Although RA has been studied for many years as a typical rheumatism, the pathogenesis are still remained to be clarified Currently, the pathogenesis of RA are considered to be a complex involving multiple immune cells, cytokines, and signaling pathways (Mcinnes and Schett, 2011). Some research have reported that the imbalance of intestinal homeostasis in recent years, including activation of intestinal mucosal immunity, disturbance of intestinal microbiome and epithelial barrier damage, may be involved to the process of RA (Bennike et al., 2017; Kayama and Takeda, 2020). Targeting intestinal immunity and barrier may become a new means to intervene in RA process (Castro-Dopico and Clatworthy, 2019; Matei et al., 2021). However, there is still a lack of systematic analysis on lymphocyte synthesis sites in RA intestinal immunity, especially after different administration times of drug in the previous research.

The total glucosides of paeony (TGP) are the effective parts extracted from the dried roots of *Paeonia lactiflora* Pall (a traditional Chinese herbal medicine), which are mainly consist of monoterpene glycosides, such as paeoniflorin, albiflorin, benzoylpaeoniflorin, oxypaeoniflorin, galloylpaeoniflorin, etc (Jiang et al., 2020). Pharmacological studies have shown that TGP has immunomodulatory effect and can intervene in the process of RA by inhibiting immune cell activation and inflammatory signals (Zhang L. et al., 2019). In clinical, TGP capsule (trade name: Pa-Fu-Lin) has been widely used to treat RA (Chen et al., 2019) and other autoimmune diseases. However, the compounds in TGP has low bioavailability and are easy to accumulate in the gastrointestinal tract (Fei et al., 2016); the most main components of TGP are excreted with feces and are not easily absorbed into the blood (Zuo et al., 2017). In our previous study, oral administration of TGP could partially reverses collagen-induced arthritis (CIA) rats dysbiosis in intestinal microbiome and regulate the levels of intestinal cytokines SIgA and IFN-γ (Peng et al., 2019). Therefore, as the immune site directly contacted by TGP after oral administration, TGP may also participate in the regulation of the intestinal immune system in RA.

Peyer’s patches (PPs), intraepithelial lymphocytes (IELs), and lamina propria lymphocytes (LPLs) are all key effector sites of the intestinal mucosal immune system. LPLs mainly include CD4^+^ T lymphocytes and B lymphocytes, which mainly secrete Th2 cytokines and IgA (Husband et al., 1996); IELs mainly include CD8^+^ T lymphocytes, which mainly play cytotoxicity and express Th1 and Th2-related cytokines (Yoshikai, 1999). Therefore, it is of great significance to detect the changes of T lymphocytes in different parts at the same time to systematically reflect the intestinal mucosal immunity, but there are few studies on this aspect at present. In this study, we simultaneously evaluated the number of T cells at three lymphocyte synthesis sites (including PPs, IELs and LPLs). The protective effect of TGP on intestinal epithelial barrier was also observed. Finally, the integrated analysis of network pharmacology and untargeted metabolomics was used to identify potential biomarkers, screen and verify the combined target of TGP in the intestine, to clarify the molecular mechanism of TGP immune regulation and protection of epithelial barrier.

## 2 Materials and methods

### 2.1 Chemicals and reagents

The total glucosides of paeony capsules were purchased from Ningbo Lihua Pharmaceutical Co., Ltd (National Drug Standard Z43020138, batch number: 210502). The methotrexate tablets was purchased from Shanghai Xinyi Pharmaceutical Co., Ltd (National Pharmaceutical Standard H31020644, batch number: 20210124). Bovine type II collagen, complete Freund’s adjuvant, and incomplete Freund’s adjuvant were purchased from Chondrex (Redmond, WA, USA). EDTA decalcification solution and Hematoxylin and eosin (H&E) staining solution were purchased from Solarbio (Beijing, China) and Servicebio (Wuhan, Hubei, China), respectively. EDTA-Na_2_, HEPES, Type IV collagenase digest, Dispase II, DNase I, D-Hank’s, and Hank’srequired for the isolation of small intestinal lymphocytes were obtained from Solarbio (Beijing, China).

The antibodies used are shown as following: Rat CD3 FITC G4.18, CD4 APC OX-35, and CD8a PE OX-8 (BD Pharmingen, Franklin Lake, NJ,USA); Mouse IgG3 KpaItCl FITC A112-3, IgG2a KpaItCl APC G155-178, and IgG1Kpa ItCl PE MOPC-31C (BD Pharmingen, Franklin Lake, NJ, USA)**.** Rat organ tissue lymphocyte separation kits were obtatined from Tianjin Haoyang Biological manufacture Co., Ltd (Tianjin, China). Primers were designed and synthesized by Beijing Ruibo Xingke Biotechnology Co., Ltd (Beijing, China).

### 2.2 Animals

Eighty healthy male Sprague-Dawley (SD) rats aged 7–8 weeks, were purchased from Beijing WeitongLihua Laboratory Animal Technology, weighing 180 ± 20 g. The animal protocol was approved by the Animal Experiment and Laboratory Animal Management Committee of Capital Medical University (ethics approval number: AEEI-2020–002), and all experimental procedures are in compliance with the US “Guidelines for the Management and Use of Laboratory Animals”. The rats were raised in the Experimental Animal Center of Capital Medical University and placed in an environment with controlled temperature and humidity, free of specific pathogens (SPF). Rats have free access to sterilized food and water. Before the experiment, the rats were kept for 7 days to adapt to the environment. Then the rats were divided into 2 groups: 16 rats in the control group and 64 rats in the CIA model development.

### 2.3 Induction of CIA and drug administration

**FIGURE 1:**
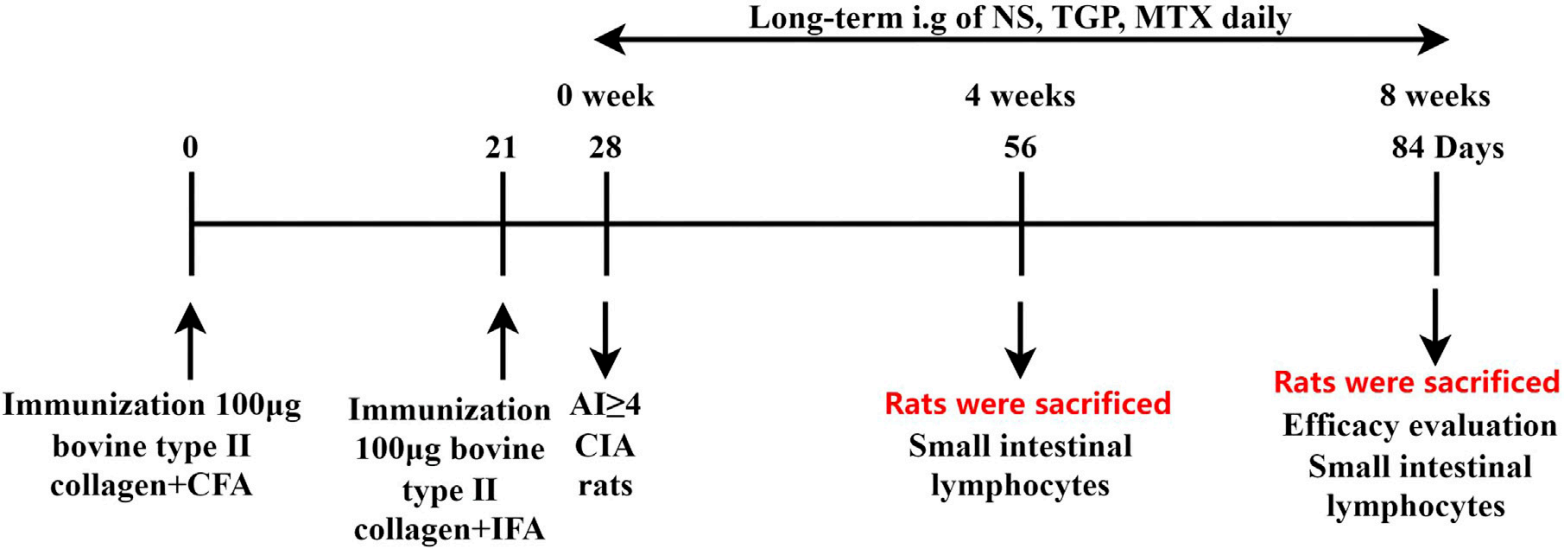
Experimental schedule. Rats were sacrificed on days 56 and 84, and their small intestinal lymphocytes were isolated and collected. All efficacy evaluation were performed on 84-day sacrificed rats. All rats were given NS, TGP, and MTX daily respectively, and pharmacodynamic evaluation was carried out at the study endpoint.

Sixty-four SD rats were induced with a 100 µL subcutaneous injection of 1 mg/mL bovine type II collagenemulsified with complete Freund’s adjuvant. On day 21, rats also received a 100 µL subcutaneous booster dose of 1 mg/mL bovine type II collagen emulsified with incomplete Freund’s adjuvant. After the induction of inflammation, the degree of joint disease was observed and recorded. According to a 5-point scale, the Arthritis index (AI) was calculated as follows: 0 = noevidence of erythema and swelling; 1 = mild erythema and swelling of the wrist or ankle; 2 = moderate erythema and swelling from the wrist to the metacarpal joints or from the ankle to the metatarsal joints; 3 = severe erythema and swelling of the entire paw including the digits and 4 = maximal erythema and swelling of the paw, or ankylosis of the limb. The sum of the scores for each of the four limbs was regarded as the total AI. 7 days later, rats with an AI ≥ 4 were regarded as successful model (Marcińska et al., 2016). According to the AI scores, and body weight, the CIA rats were randomly divided into 4 groups (n = 16): a model group, a CIA + low-dose CIA TGP group (TGPL), a CIA + high-dose CIA TGP group (TGPH), and a CIA + methotrexate group (MTX). The low and high doses of TGP were 158 mg/kg (a clinical equivalent dose), 316 mg/kg (2 times the clinical equivalent dose), respectively. The dose of methotrexate was 0.88 mg/kg (a clinical equivalent dose). The control group and model group were given equal volumes of normal saline (NS). All of the drugs were administered continuously for 8 weeks. One-half of the rats were sacrificed at 56 days and the remaining rats were sacrificed at 84 days. The experimental schedules are shown in Figure 1.

### 2.4 Physiological parameters

Three physiological indicators were used to evaluate the development of the CIA model and the overall efficacy of TGP: body weight, posterior plantar thickness, and AI. After the secondary immunization of rats was completed, the body weight, degree of foot swelling, and AI were evaluated every 7 days. The degree of foot swelling was determined by the average thickness of the left and right hind paws of rats.

### 2.5 Hematological analyses

After 8 weeks of administration, the remaining 40 rats were anesthetized with isoflurane, then blood was collected in a heparin sodium tube, and the left hind limb was amputated at 1 cm above the ankle joint. After centrifuging, the supernatant was collected, and the commercial enzyme linked immunosorbent assay (ELISA) kits were used to measure the concentration of C-reactive protein (CRP) (Jiancheng Bioengineering Institute, Nanjing, Jiangsu, China) and anti-Collagen II antibody (anti-CII) (Fankew, Shanghai, China) in plasma according to the instructions.

### 2.6 Histological examination of joints and bone microarchitecture

The hind paws of rats were taken out after death, stored in 4% paraformaldehyde solution, decalcified in 20% EDTA for 6 weeks, then dehydrated and embedded in paraffin. Next, H&E staining was used to examine changes in the ankle joint. Histological examination was performed by blind method for cartilage damage, synovial hypertrophy and changes in inflammatory cell infiltration, and graded from 0–3 as previously described (Helyes et al., 2004; Larsson et al., 2004). Furthermore, the left paw and the distal end of the left femur (already amputated) were vertically fixed along the long axis to the small animal Inveo PET/CT/SPECT (Siemens, Nuremberg, Bavaria, Germany). The scan condition was as below: scanning angle was 360°; voltage was 80 kv; current was 500 μA; resolution was 14.93 μm.

### 2.7 Flow cytometry

At 56 and 84 days respectively, the rats were excessively anesthetized and sacrificed. The abdominal cavity of rats was opened to remove the entire small intestine from the pylorus to the cecum, and the mesenteric fat and intestinal contents were removed. The lymphocytes of Peyer’s patches (PPs) were obtained by mechanical grinding method (Qiu and Sheridan, 2018), the intestinal intraepithelial lymphocytes (IELs) were obtained by EDTA dissociation method (Benakis et al., 2016), and the lamina propria lymphocytes (LPLs) were obtained by enzymatic digestion method (Mcmanus et al., 2019). In accordance with the instructions for use of the rat organ tissue lymphocyte separation solution, the IELs and the LPLs were separated from the above single cells by density gradient centrifugation.

Each part of lymphocytes obtained by the above separation was adjusted to a cell suspension with a cell concentration of 5×10^6^/mL with PBS containing 2% NBS. The lymphocytes isolated above were subjected to immunofluorescence staining with Rat FITC, APC, PE (BD Pharmingen, USA), and labeled anti-rat CD3^+^, CD4^+^, and CD8^+^ monoclonal antibodies, respectively, reacting at 4°C for 30 min in the dark. Subsequently, the labeled cell suspension was washed twice and resuspended in PBS. The resuspension was filtered through a 300 mesh cell screen and analyzed with BD LSRFortessa custom flow cytometer (Becton, Dickinson and Company, Franklin Lake, NJ, USA).

### 2.8 Histological evaluation of small intestine epithelial barrier

The small intestine was removed, and the part near the cecum was collected about 1 cm. The samples were placed in 4% paraformaldehyde solution at room temperature for 48 h to fix for H&E staining. In brief, the fixed small intestine tissue was embedded in paraffin and cut into 10 μm serial sections. These tissue sections were dewaxed with xylene, then gradient eluted in alcohol and dehydrated. Subsequently, sections were stained with hematoxylin for 5 min at room temperature and counterstained with eosin for 5 min, which were scaned by a panoramic scanner and visualized by CaseViewer 2.4. Subsequently, The villus length and the crypt depth were measured and the ratio of villi length to crypt depth (V/C) was calculated.

### 2.9 Immunohistochemistry and immunofluorescence analysis

Immunohistochemistry was also performed to detect immunoreactivity for CD4, CD8, ZO-1 and occludin in the small intestine. These assays used a primary ZO-1 (1:1,000; GB111402, Servicebio, China) and occludin (1:1,000; GB111401, Servicebio, China) antibody and a goat anti-rat IgG as secondary antibody (K5007, DAKO, Denmark). Positive signals were detected with 3, 3′-diaminobenzidine (DAB) (G1211, Servicebio, China). Slides were counterstained with hematoxylin (G1040, Servicebio, China) and positive cells identified by the presence of brown particles. Five randomfields were evaluated from each section and Image-Pro Plus 6.0 software (Media Cybernetics Inc., MD, USA) was used to quantify the proportion (in %) of ZO-1 and occludin-positive cells.

For immunofluorescence, after deparaffinization, tissue sections were incubated with anti-rabbit ADA (1:400; GB112099, Servicebio, Wuhan, China) and PNP (1:300; GB113688, Servicebio, Wuhan, China) primary antibody and conjugated FITC goat anti-rabbit antibody (1:400) for overnight. Then, DAPI (2 μg/mL) was used for nucleus staining. The images were taken by a fluorescence microscope (Nikon Eclipse Ci-L, ×200 magnification). The relative mean fluorescence intensity of ADA and PNP-secreting cells were counted by Image-Pro Plus 6.0 software.

### 2.10 Real-time quantitative PCR assays

The flash-frozensmall intestine tissues were processed according to the instruction manuals, and the mRNA expression of target genes (ZO-1, occludin, ADA and PNP) were determined. The total RNA was extracted using total RNA kit (W0214, TIANGEN, China) and then the isolated RNA was reverse-transcribed into cDNA with FastkKing cDNA synthesis kit (W0008, TIANGEN, China). RT-qPCR analysis was performed on the PCR (Bio-Rad, Hercules, CA, USA). The specific PCR amplification parameters are as follows: a 3 min pre-denaturation at 95°C and 40 cycles (95°C for 10 s, 60°C for 10 s, and 72°C for 30 s). The primer sequences used are shown in the Supplementary Table S1. The levels of target mRNA expression were normalized based on the level of the reference gene β-actin, and the results were calculated with the 2^−ΔΔCt^ method.

### 2.11 Samples collections and preparation for metabolomics analysis

Each small intestine samples were ground into powder by adding liquid nitrogen in a mortar, and then placed in a vacuum freeze dryer (Marin Christ, Osterode, Germany) for 24 h to remove moisture. 20 mg of lyophilized small intestine was placed into a 2 mL centrifuge tube. Then 500 μL of pre-cooled methanol-acetonitrile-water (5:3:2, *v*/*v*/*v*) and 5 zirconium beads were added in the centrifuge tube, and homogenizing these samples for 3 min in the cryogenic tissue homogenizer (Q24RC, Dinghaoyuan Technology, Tianjin, China). The mixture was then vortexed for 2 min and then sonicated in an ice-water bath for 5 min 400 μL supernatant was collected after a centrifugation at 14,000 rpm for 10 min under 4°C and dried in gentle N_2_ flow at 25°C. The dried residue was reconstituted by adding 300 μL of methanol, vortexed for 1 min, and centrifuged at 14,000 rpm/min, and then 4 μL of the supernatant was analyzed by LC-MS. A quality control (QC) sample which was prepared by mixing equal volumes of each sample was used.

### 2.12 UHPLC-Q-extractive MS conditions

The small intestinal tissue extracts were analyzed on the UltiMate 3000 RS UHPLC Systems (Thermo Fisher Corporation, Milford, MA, USA), equipped with a Waters ACQUITYUPLC UPLC BEH C_18_ column (100 mm × 2.1 mm, 1.7 µm, Waters, Milford, CT, USA) maintained at 40°C. The flow rate of mobile phase was 0.3 mL/min, which was constituted by solvents A (H_2_O+0.1% acetic acid) and B (Acetonitrile+0.1% acetic acid). The gradient elution was shown as following program: 0–2 min, 5%B; 2–6 min, 5–50%B; 6–11 min, 50–60%B; 11–20 min, 60–95%B; 20–23 min, 95%B; 23–23.1, min 95–5%B and balanced with 5% B for 3 min. Both the positive and negative ion modes of mass detection were used in mass spectrometry using the electrospray ionization (ESI) source in the Q Exactive HF MS (Thermo FisherCorporation, Milford, MA, USA). The specific parameters were as below: scan mode was full scan; scan range: m/z 70–1,000; primary resolution was 120,000, secondary resolution was 30,000; fragmentation energy was set to 20, 40, 60 eV; the sheath gas flow rate was 45 psi and the auxiliary gas flow rate was 15 psi; ion transfer tube temperature was 320°C, and auxiliary gas heating temperature was 350°C. The QC was inserted every 8 samples to evaluate the stability of the instrument operation.

### 2.13 Data processing for metabolomics

All total ion chromatograms of LC-MS were exported by Xcalibur workstation (Thermo Fisher Corporation, Milford, MA, USA) and imported into Compound Discovery 3.2 software (Thermo Fisher Corporation, Milford, MA, USA) for peak extraction, normalization and alignment. The obtained data were then imported into SIMCA-P 14.1 (Umetrics, Umea, Sweden) for multivariate statistical analysis. Principal component analysis (PCA) was used to first describe the metabolites in the small intestinal tissue globally. Orthogonal least squares discriminant analysis (OPLS-DA) was then used for classification discrimination to obtain variable importance (VIP) in projections of metabolites. Metabolites satisfying VIP >1, *p* < 0.05, and log_2_ Fold Change (FC) > 1 or < −1 were screened as differential metabolites. HMDB (http://www.hmdb.ca/spectra/ms/search), KEGG (http://www.genome.jp/kegg/), BioCyc (https://www.biocyc.org/), and m/z Cloud (https://www.mzcloud.org/) were used for metabolite identification. To analyze the metabolic networks involved in differential metabolites, metabolic pathway analysis was performed using MetaboAnalyst 5.0 (http://www.metaboanalyst.ca/). TGP-regulated differential metabolites were imported into MetScape, a plugin within the Cytoscape software (version 3.8.0), to build component-reaction-gene-enzyme networks and capture relevant targets.

### 2.14 Network pharmacology analysis

Based on our previous characterization of the TGP capsule contents, ten major monoterpenes and their glycosides components were selected as candidate molecules for further analysis (Supplementary Data Sheet S2). The SMILES structures of the above molecules were imported into the databases SwissTargetPrediction (http://www.swisstargetprediction.ch/index.php) and Similarity Ensemble Approach (http://sea.bkslab.org/) to obtain the corresponding targets. RA-related gene targets were subsequently obtained from the database Therapeutic Target Database (http://db.idrblab.net/ttd/), DisGeNET (https://www.disgenet.org/) (the correlation score >0.05) and Drugbank (https://go.drugbank.com/) (the correlation score >0.5). After the collected targets are merged and duplicates removed, potential targets of TGP against RA were obtained by crossing TGP-associated gene targets with RA-associated gene targets.

The intersection targets were imported into the String (https://string-db.org/) website to get a protein-protein interaction (PPI) network diagram. Then, CytoHubba plug-in of Cytoscape 3.8.0 was used to screen the top ten joint targets based on the MCC algorithm. The Gene Ontology (GO) and Kyoto Encyclopedia of Genes and Genomes (KEGG) enrichment analysis of potential targets was performed using the DAVID database (https://david.ncifcrf.gov/). All results are visualized by bioinformatics online platform (http://www.bioinformatics.com.cn).

### 2.15 Molecular docking

First, based on the results of integrated analysis of metabolomics and network pharmacology, key targets and TGP-related components were identified. Secondly, the structure of the key targets were obtained from the protein database (PDB, https://www.rcsb.org/), and the 3D structure of the ligands were downloaded from PubChem (https://pubchem.ncbi.nlm.nih.gov/). Subsequently, the structures of ligands and receptors were imported into AutoDock Tools 1.5.7 for preprocessing. Ligands are charged and automatically recognize torsional bonds. The receptors are stripped of the original ligand and water molecules, then added hydrogen atoms. Both ligands and receptors are exported as PDBQT format files. Finally, the AutoDock Vina was used to determine the extent of the active pocket and perform molecular docking. Visualization of docked objects was performed by PyMOL 2.4.1.

### 2.16 Statistical analysis

Data were derived from three independent experiments and expressed as mean ± standard (SD) deviation. Statistical analysis was carried out using student’s paired *t*-test and one-way analysis of variance (ANOVA), in order to obtain the differences between the groups. The data of body weight and foot thickness were analyzed by repeated measurement analysis of variance. All analyses were performed using the GraphPad Prism v8.0 software (GraphPad Software Inc, San Diego, CA, USA). A difference of *p* < 0.05 was deemed as statistically significant.

## 3 Results

### 3.1 Physiological parameters

**FIGURE 2:**
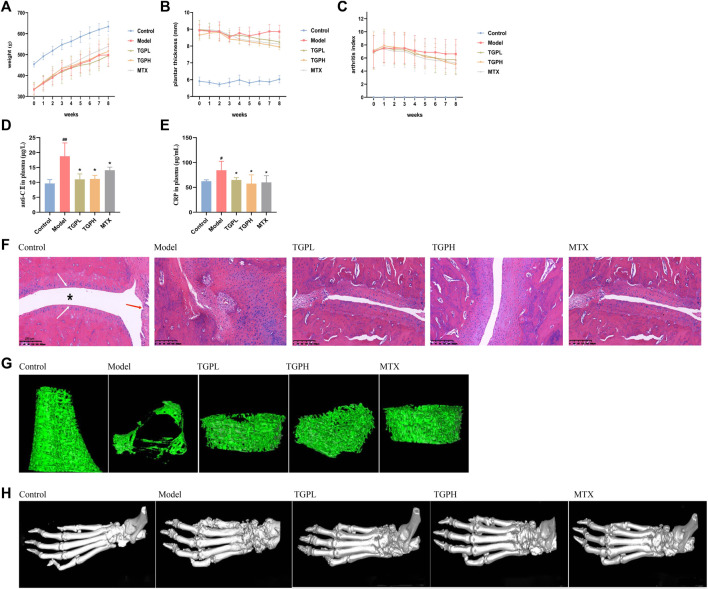
Pharmacodynamic evaluation of TGP on CIA rats. **(A)** body weight, **(B)** hindfoot thickness, and **(C)** AI (n = 8). **(D)** Anti-collagen II antibody (anti-CII) in the rats plasma. **(E)** C-reactive protein (CRP) in the rats plasma. All data are expressed as mean ± SD (n = 5), ^#^ *p* < 0.05, ^##^ *p* < 0.01 vs. control group; ^*^ *p* < 0.05 vs. model group. **(F)** Representative sections of histopathologic results of ankle joints. The position of “*” represents the joint capsule, the white arrow indicates the articular cartilage, and the blue arrow indicates the normal synovial cells. Magnifcation ×100. Three-dimensional visualization of Micro-CT imaging of trabecular bone **(G)** and foot joints **(H)** in all groups of rats.

After the CIA model was successfully constructed, the rats showed symptoms such as reduced activity, weight loss, stiffness of the hind limbs, and swelling of the foot joints. The body weight, hind paw thickness and AI of rats in all groups are shown in Figures 2A–C. Compared with the Model group, the TGPL and TGPH groups had no significant effect on the body weight of CIA rats, while the MTX group’s weight growth rate increased slowly from the third week of administration. In addition, TGP and MTX showed an alleviating effect on the swelling of the hind paws of CIA rats starting from the fourth week. This trend could also be observed on the AI of CIA rats.

### 3.2 Quantification of plasma Anti-CII and CRP

Anti-CII and CRP are considered to be closely related to the joint lesions of RA and are used as blood markers for early diagnosis of RA (Manivel et al., 2015; Kaplan et al., 2023). ELISA results showed that the levels of anti-CII and CRP in the plasma of CIA rats were significantly increased (*p* < 0.01) compared with the control group. After the administration of TGP, anti-CII and CRP were significantly decreased in TGPL (*p* < 0.05, *p* < 0.05), TGPH (*p* < 0.05, *p* < 0.05) and MTX (*p* < 0.05, *p* < 0.05) (Figures 2D,E).

### 3.3 Histopathology and Micro-CT analysis of joint and bone

The histopathological results of rat joints were shown in Figure 2F. In normal rats, the articular surface was covered with hyaline cartilage, the joint capsule structure was clear, and the synovial cells were continuous. In CIA rats, the articular surface cartilage was eroded, the joint capsule was narrowed, the synovial tissue was proliferated, and a large number of inflammatory cells were infiltrated. After administration, TGP and positive drugs can protect articular cartilage, inhibit synovial tissue proliferation, and reduce inflammatory cell infiltration to a certain extent. The micro-CT results of joints and bone were shown in Figure 2G. Compared with the control group, the trabecular bone of the distal femur of the rats in the model group was sparse, the structure was disordered, the distance between the trabecular bone was increased, and the microstructure was significantly damaged. In the group of TGPL, TGPH, and MTX, it was observed that the number of the femoral trabeculae of rats was decreased, the distance between trabeculae increased slightly, and the integrity of the microstructure was basically maintained. The 3D visualization imaging results of Micro-CT of foot joint morphology are shown in Figure 2H. Compared with the control group, the articular surface of the model group was rough and the joint structure was blurred. The TGP administration group also showed that the ankle joint had a certain degree of bone destruction, but there was decreasing trend compared with the model group.

### 3.4 Regulation of TGP on intestinal mucosal immunity system in CIA rats

In order to simulate the 4-week onset of TGP clinical administration and the need for long-term administration, we also tested the effects of TGP administration on intestinal immunity for 4 weeks and 8 weeks. The flow cytometry was used to detect the levels of CD3^+^, CD4^+^ and CD8^+^ cells in different positions of the intestinal mucosal immune system and calculated the number of PPs, so as to observe the changes of intestinal mucosal immunity in CIA rats and the effect of TGP at different administration times.

**FIGURE 3:**
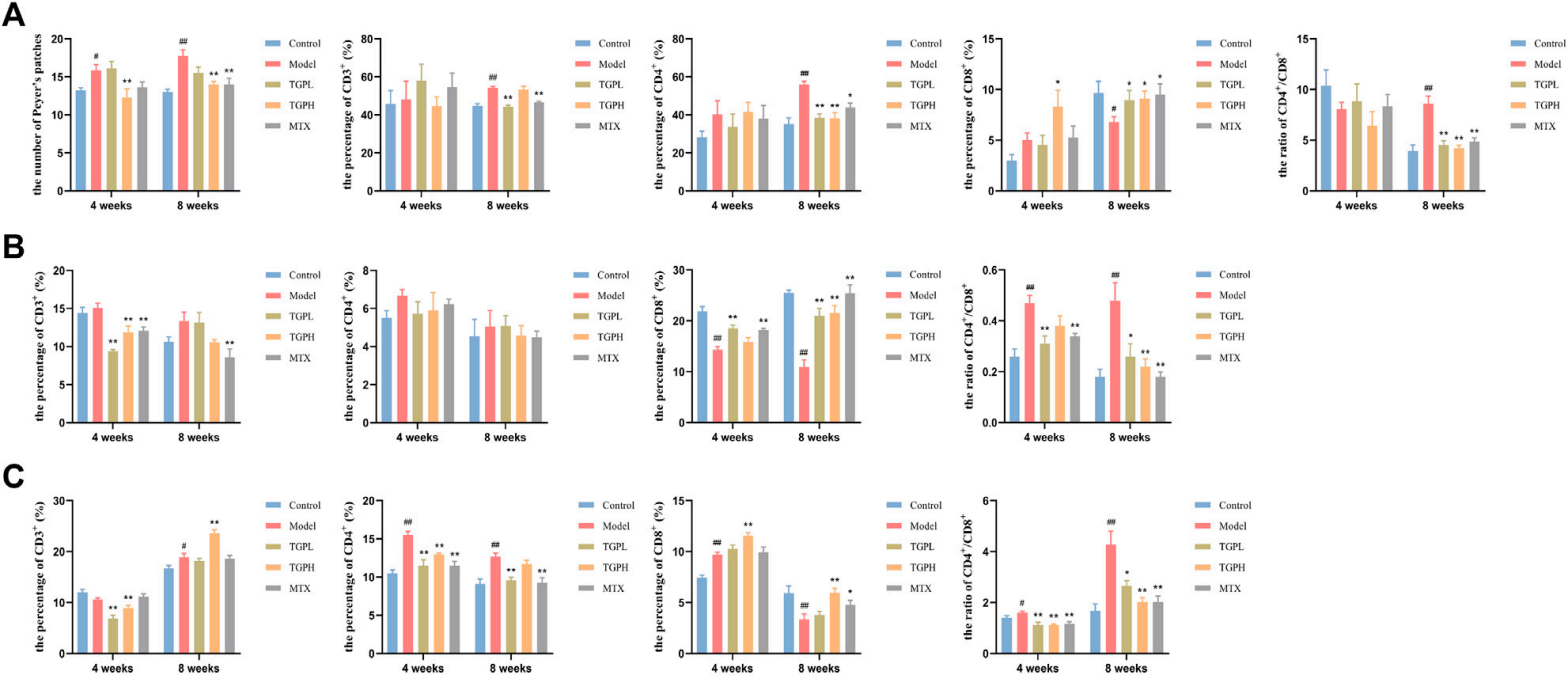
Effect of TGP on the intestinal mucosal immunity (n = 5–6). **(A)** The count of PPs and the percentage of CD3^+^, CD4^+^, CD8^+^ cells and CD4^+^/CD8^+^ ratio in PPs lymphocytes. **(B)** The percentage of CD3^+^, CD4^+^, CD8^+^ cells and CD4+/CD8+ ratio in LELs. **(C)** The percentage of CD3^+^, CD4^+^, CD8^+^ cells and CD4^+^/CD8^+^ ratio in LPLs. ^#^ *p* < 0.05, ^##^ *p* < 0.01 vs. control group; ^*^ *p* < 0.05, ^**^ *p* < 0.01 vs. model group.

As shown in Figure 3A and Supplementary Figure S1, at 4 weeks of administration, compared with the control group, the number of PPs in the model group was significantly reduced (*p* < 0.05), while only high-dose TGP could significantly reduce the number of PPs in CIA rats (*p* < 0.01). When the dosing time was increased to 8 weeks, in addition to TGPH (*p* < 0.01), the number of PPs in MTX was also significantly decreased (*p* < 0.01). At 4 weeks of administration, compared with the control group, the percentage of CD3^+^, CD4^+^, CD8^+^ cells and the CD4^+^/CD8^+^ cells ratio were not significantly different in the model group; only the percentage of CD8^+^ cells was significantly increased in TGPL compared with the model group (*p* < 0.05). When the administration time was increased to 8 weeks, compared with the model group, the percentage of CD4^+^ cells and the CD4^+^/CD8^+^ cells ratio were significantly decreased, and the percentage of CD8^+^ cells was significantly increased in TGPL (*p* < 0.01, *p* < 0.01, *p* < 0.05), TGPH (*p* < 0.01, *p* < 0.01, *p* < 0.05), and MTX (*p* < 0.05, *p* < 0.01, *p* < 0.05). However, compared with TGPL (*p* < 0.01) and MTX (*p* < 0.01), TGPH did not show a significant effect on CD3^+^ cells.

Figure 3B and Supplementary Figure S2 showed the effect of TGP on the LELs. At 4 weeks of administration, compared with the control group, the model group first showed a significant decrease in the percentage of CD8^+^ cells (*p* < 0.01) and a significant increase in the ratio of CD4^+^/CD8^+^ cells (*p* < 0.01). Compared with the model group, TGPL (*p* < 0.01, *p* < 0.05, *p* < 0.01) and MTX (*p* < 0.01, *p* < 0.01, *p* < 0.01) showed a significant decrease in the percentage of CD3^+^ cells and the CD4^+^/CD8^+^ cells ratio, and the percentage of CD8^+^ cells was significantly increased. However, TGPH (*p* < 0.01) only showed a significant decrease in the percentage of CD3^+^ cells. When the administration time was increased to 8 weeks, compared with the control group, the model group still did not show a significant difference in the percentage of CD3^+^ and CD4^+^ cells. Compared with the model group, the percentage of CD8^+^ cells was significantly increased and the CD4^+^/CD8^+^ cells ratio were significantly decreased in TGPL (*p* < 0.05, *p* < 0.01), TGPH (*p* < 0.01, *p* < 0.01), and MTX (*p* < 0.01, *p* < 0.01). Interestingly, all groups did not show significant differences in the percentage of CD4^+^ cells regardless of the 4 weeks or 8 weeks of administration.

Figure 3C and Supplementary Figure S3 showed the effect of TGP on the LPLs. At 4 weeks of administration, compared with the control group, the model group showed a significant increase in the percentage of CD4^+^, CD8^+^ cells and the CD4^+^/CD8^+^ cells ratio (*p* < 0.01, *p* < 0.01, *p* < 0.05). Compared with the model group, both TGPL (*p* < 0.01, *p* < 0.01, *p* < 0.01) and TGPH (*p* < 0.01, *p* < 0.01, *p* < 0.01) could significantly decrease the percentage of CD3^+^, CD4^+^ cells and the CD4^+^/CD8^+^ cells ratio in CIA rats, but only in TGPH (*p* < 0.01) showed a significant increase in the percentage of CD8^+^ cells. Different from TGP, MTX (*p* < 0.01, *p* < 0.01) only had inhibitory effect on the percentage of CD4^+^ cells and CD4^+^/CD8^+^ cells ratio. When the administration time was increased to 8 weeks, the difference from 4 weeks was that the percentage of CD3^+^ cells (*p* < 0.05) was significantly increased and the percentage of CD8^+^ cells (*p* < 0.01) was significantly decreased in the model group compared with the control group. TGPL (*p* < 0.05), TGPH (*p* < 0.01), MTX (*p* < 0.01) all showed a significant decrease in the CD4^+^/CD8^+^ cells ratio, but there were difference in modulating the percentage of CD3^+^, CD4^+^, CD8^+^ cells.

These results suggested that TGP could systematically reverse the imbalance of CD4^+^/CD8^+^ in different parts of small intestinal immune system in CIA rats, so as to maintained intestinal immune homeostasis. In general, the effects of TGP on LELs and LPLs started mainly from 4 weeks of administration, while the effects on PPs lymphocytes started mainly from 8 weeks of administration. Interestingly, there were differences in the onset time of lymphocytes in different parts at different doses. Compared with TGPL, TGPH showed an earlier effect in regulating the number of PPs and the immune function of PPs lymphocytes. However, TGPL showed an earlier effect in regulating IELs.

### 3.5 The regulatory effect of TGP on the small intestinal epithelial barrier

**FIGURE 4:**
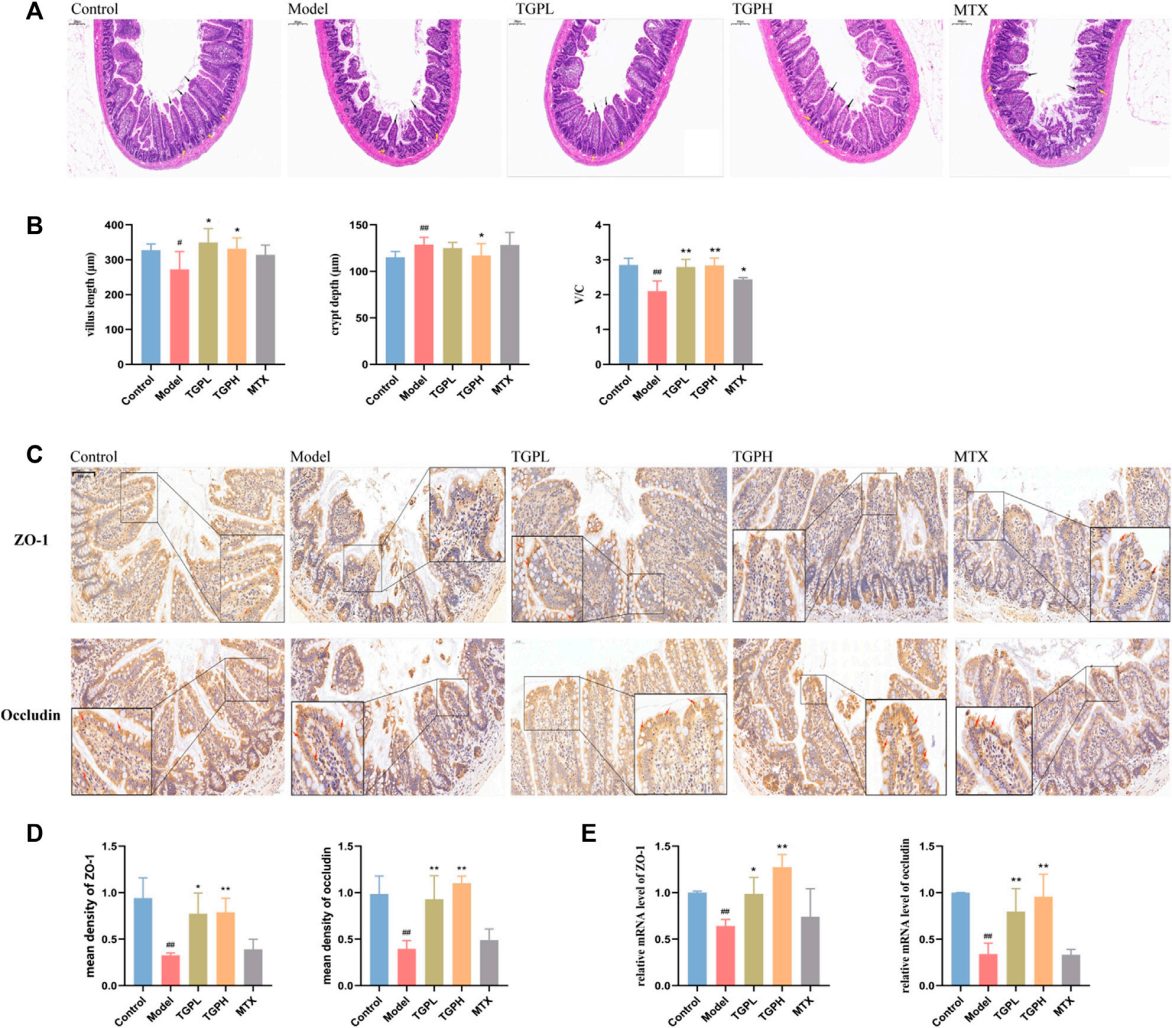
Effects of TGP on the small intestinal epithelial barrier. **(A)** Representative small intestine HE-stained images and **(B)** measurement and calculation of villus length, crypt depth, and V/C (n = 6) (scale bars = 200 μm). **(C)** Representative immunohistochemistry images and **(D, E)** levels of ZO-1 and occludin in small intestine (scale bars = 50 μm) (n = 4). ^#^ *p* < 0.05, ^##^ *p* < 0.01 vs. control group; ^*^ *p* < 0.05, ^**^ *p* < 0.01 vs. model group.

Compared with the control group, the intestinal villus length (*p* < 0.05) and the ratio of V/C (*p* < 0.01) in the model group were significantly decreased, and the crypt depth (*p* < 0.01) was significantly deepened, which indicated that the intestinal barrier of the rats was damaged after the successful CIA modeling. After 8 weeks of administration, both the lowand high doses of TGP increased the intestinal villus length (*p* < 0.05) and the ratio of V/C (*p* < 0.01), and the high dose of TGP was found to significantly reduce the depth of crypts (*p* < 0.05) in CIA rats (Figure 4A). H&E staining results were shown in Figure 4B. The intestinal villi of rats in normal state were neatly arranged, compact and complete. However, the morphology of the intestinal villi of CIA model rats was obviously broken, atrophied and shortened. After TGP treatment, the rat intestinal villi had obvious recovery, and the symptoms such as breakage and atrophy were alleviated to varying degrees, and the villus structure was gradually restored and arranged neatly. However, MTX administration did not improve the damaged state of intestinal epithelial barrier. Subsequently, RT-qPCR and immunohistochemistry were used to determine the content of two Tight junctions (TJ) proteins (ZO-1 and occludin) in the small intestine for the assessment of intestinal epithelial permeability. The results of immunohistochemistry showed that the protein expressions of ZO-1 (*p* < 0.01) and occludin (*p* < 0.05) in the model group were significantly decreased compared with the control group. However, both TGPL (*p* < 0.05, *p* < 0.05) and TGPH (*p* < 0.01, *p* < 0.01) significantly increased the expression of ZO-1 and occludin compared with the model group (Figures 4C,D). RT-qPCR was conducted and the results of gene expressions of ZO-1 and occludin were consistent with those in immunohistochemistry (Figure 4E).

### 3.6 Differential metabolites in the small intestine

QC samples are used to evaluate the technical precision and reproducibility of analytical batches and as an indicator for data quality assessment, and the relative standard deviation (RSD) of each metabolite peak area was calculated. The results are shown in Supplementary Figure S4A, metabolite peaks area with RSD% less than 30% in positive and negative ion mode are 97.05% and 82.64%, respectively**.** As shown in Supplementary Figure S4B, C, all 7 QC samples are within ± 2SD range of the score plot. The above results indicated that the developed LC-MS method is stable and reliable, and the quality of metabolomics data can be guaranteed.

**FIGURE 5:**
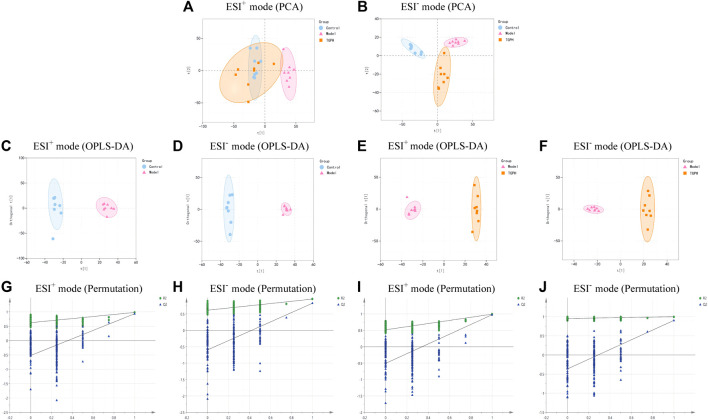
PCA score plots base on the LC-MS data of control group, model group and high-dose CIA TGP group (TGPH) in positive **(A)** and negative **(B)** ion models (n = 8). OPLS-DA analysis between model and control groups in positive **(C)** and negative **(D)** ion models and the corresponding permutation test plots **(G, H)** (n = 8). OPLS-DA analysis between model and TGPH groups in positive **(E)** and negative **(F)** ion models and the corresponding permutation test plots **(I, J)**.

**FIGURE 6:**
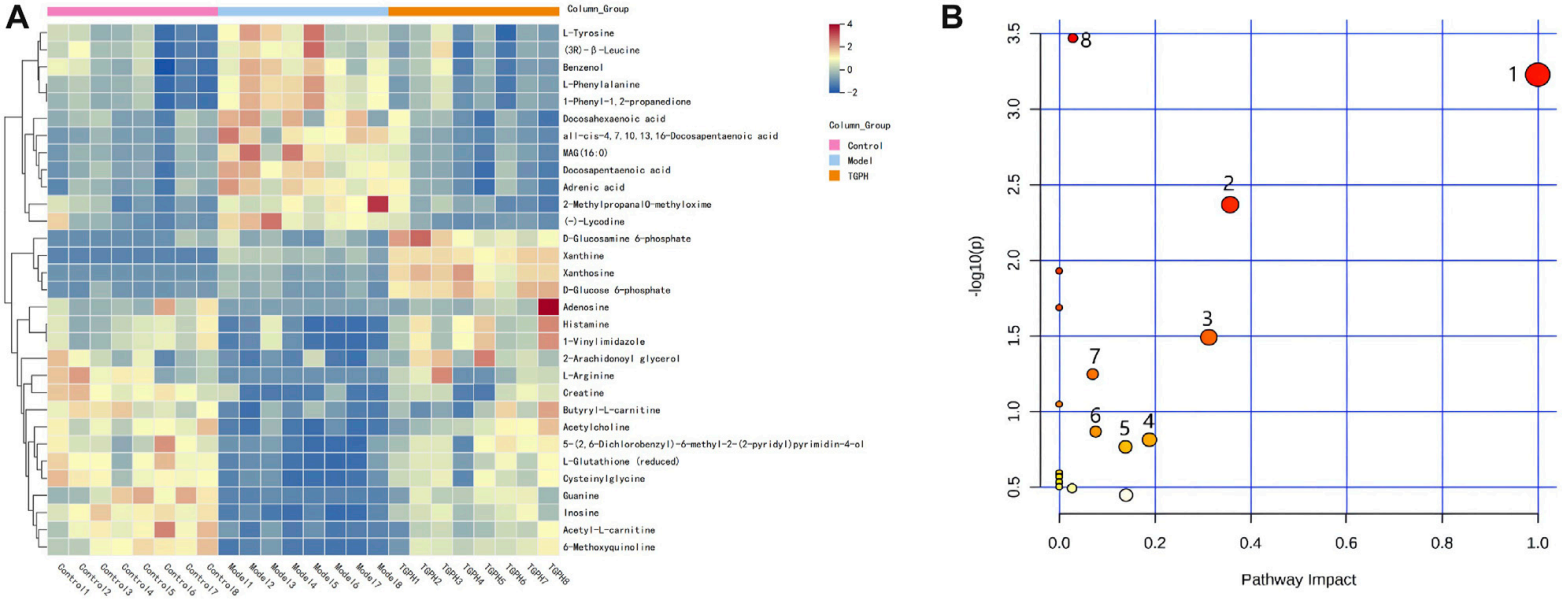
**(A)** Differential metabolites of heatmap related to intervention of TGPH. Red area: an increase of up to two folds; blue area: a reduction of up to two folds. **(B)** Network enrichment analysis of metabolic pathway associated with TGPH. 1. phenylalanine, tyrosine and tryptophan biosynthesis; 2. phenylalanine metabolism; 3. glutathione metabolism; 4. histidine metabolism; 5. starch and sucrose metabolism; 6. arginine biosynthesis; 7. arginine and proline metabolism; 8. purine metabolism.

According to the efficacy evaluation results, the overall treatment effect of high-dose TGP is better than that of low-dose TGP, therefore the control group, model group and TGPH were selected for untargeted metabonomics analysis. The PCA score plots show that the model group was significantly separated from the control group, it suggests that modeling significantly changed the metabolic profile of small intestine tissue. While, TGPH can make the metabolic wheel contour of CIA rats migrate to the control group, indicating that TGP has a certain regulatory effect on the metabolic disorder in the small intestine of CIA rats (Figures 5A,B). The difference compounds are initially screened out through the condition of VIP >1, log_2_ FC > 1 or < −1, and then the ones that meet the condition of *p* < 0.05 are screened out through the *t*-test in OPLS-DA (Figures 5C–F). Subsequently, permutation tests were utilized to validate the established OPLS-DA model. The *R* ^2^ and Q^2^ values of the model were good after 200 response permutation tests, indicating that the OPLS-DA model was reliable without a overfitting (Figures 5G–J). Between the control group and the model group, a total of 62 metabolites were identified as potential biomarkers, including 32 positive ion modes and 30 negative ion modes. It was found that 32 specific metabolites were related to the regulation of intestinal immunity system with TGP, and relevant information and variation trends between groups were listed in Table 1 and Table 2. The heat map was shown in Figure 6A to intuitively explain the content differences and changes between the three groups of biomarkers.

**TABLE 1:** Biomarkers associated with TGP in positive ion model (n = 8).

No.	Name	Formula	Error (ppm)	Calc. MW	RT [min]	Model/Control		TGPH/Model	
						Trend	Log_2_ FC	Trend	Log_2_ FC
1	L-Tyrosine	C_9_H_11_NO_3_	3.81	181.07458	1.007	↑^a^	1.08	↓^b^	−1.16
2	L-Phenylalanine	C_9_H_11_NO_2_	4.75	165.07976	1.077	↑^a^	1.95	↓^c^	−1.18
3	L-Histidine	C_6_H_9_N_3_O_2_	4.91	155.07024	0.897	↓^d^	−7.72	↑^c^	1.83
4	L-Glutathione	C_10_H_17_N_3_O_6_S	2.89	307.08469	1.017	↓^a^	−1.73	↑^c^	1.49
5	L (+)-Citrulline	C_6_H_13_N_3_O_3_	4.36	175.09646	0.991	↓^a^	−1.40	↑	0.8
6	Inosine	C_10_H_12_N_4_O_5_	3.94	268.08183	1.020	↓^a^	−1.83	↑^c^	1.50
7	Histamine	C_5_H_9_N_3_	8.49	111.08059	0.888	↓^a^	−2.81	↑^c^	2.75
8	Hexanoylcarnitine	C_13_H_25_NO_4_	4.16	259.17944	5.643	↓^a^	−1.33	↑	0.84
9	Guanosine	C_10_H_13_N_5_O_5_	3.34	283.09261	1.290	↓^a^	−3.47	↑	3.53
10	Guanine	C_5_H_5_N_5_O	4.00	151.05001	1.292	↓^a^	−3.49	↑^c^	3.34
11	Docosapentaenoic acid	C_22_H_34_O_2_	5.02	330.25754	18.078	↑^a^	1.08	↓^b^	−1.1
12	Docosahexaenoic acid	C_22_H_32_O_2_	4.51	328.24171	17.413	↑^a^	1.31	↓^b^	−1.15
13	L-Carnitine	C_4_H_9_N_3_O_2_	5.00	131.07013	0.986	↓^a^	−0.53	↑	0.25
14	L-Arginine	C_6_H_14_N_4_O_2_	4.42	174.11244	0.904	↓^a^	−7.82	↑^c^	7.35
15	D-1-Aminopropan-2-olO-phosphate	C_3_H_10_NO_4_P	4.96	155.03551	0.959	↓^a^	−1.64	↑	1.23
16	Cysteinylglycine	C_6_H_14_N_4_O_2_	4.42	174.11244	0.904	↓^a^	−1.85	↑^c^	1.56
17	Creatine	C_4_H_9_N_3_O_2_	5.00	131.07013	0.986	↓^a^	−7.5	↑^c^	7.07
18	Butyryl-L-carnitine	C_11_H_21_NO_4_	4.44	231.14808	2.536	↓^a^	−1.37	↑^b^	1.08
19	benzodepa	C_12_H_16_N_3_O_3_P	4.74	281.09426	1.272	↓^a^	−3.78	↑	3.75
20	all-cis-4,7,10,13,16-Docosapentaenoic acid	C_22_H_34_O_2_	5.02	330.25754	18.619	↑^a^	2.17	↓^c^	−1.86
21	Adrenic acid	C_22_H_36_O_2_	4.28	332.27295	19.262	↑^a^	1.28	↓^c^	−1.14
22	Adenosine	C_22_H_34_O_2_	5.02	330.25754	18.619	↓^a^	−1.98	↑^c^	1.57
23	Acetylcholine	C_10_H_13_N_5_O_4_	3.57	267.09771	1.277	↓^a^	−1.82	↑^c^	1.65
24	Acetyl-L-carnitine	C_9_H_17_NO_4_	4.84	203.11674	1.020	↓^a^	−1.21	↑^c^	1.03
25	6-Methoxyquinoline	C_10_H_9_NO	4.67	159.06916	1.544	↓^a^	−4.07	↑^c^	3.65
26	3-Methylsulfolene	C_5_H_8_O_2_S	2.75	132.02486	0.987	↓^a^	−6.27	↑	5.62
27	2-MethylpropanalO-methyloxime	C_5_H_11_NO	3.6	101.08443	0.997	↑^a^	1.5	↓^c^	−1.14
28	1-Vinylimidazole	C_5_H_6_N_2_	8.85	94.05393	0.892	↓^a^	−2.73	↑^c^	2.62
29	1-Phenyl-1,2-propanedione	C_9_H_8_O_2_	4.93	148.05316	1.072	↑^a^	1.03	↓^c^	−1.24
30	MAG (16:0)	C_19_H_38_O_4_	4.02	330.27834	17.696	↑^a^	1.37	↓^c^	−1.16
31	1-Deoxy-L-mannitol	C_6_H_14_O_5_	4.23	166.08325	1.783	↑^a^	2.88	↓	−1.02
32	(−)-Lycodine	C_16_H_22_N_2_	−5.26	242.1772	0.971	↑^a^	1.21	↓^c^	−1.15

^d^ *p* < 0.05.

^a^ *p* < 0.05 vs. Control group.

^b^ *p* < 0.05.

^c^ *p* < 0.01 vs. Model group.

**TABLE 2:** Biomarkers associated with TGP in negative ion model (n = 8).

No.	Name	Formula	Error (ppm)	Calc. MW	RT [min]	Model/Control		TGPH/Model	
						Trend	Log_2_ FC	Trend	Log_2_ FC
1	Xanthosine	C_10_H_12_N_4_O_6_	1.55	284.07613	1.452	↑^a^	10.69	↓^b^	−2.53
2	Xanthine	C_5_H_4_N_4_O_2_	−7.3	152.03232	1.255	↑^a^	10.33	↓^c^	−1.25
3	Taurochenodeoxycholic Acid (sodium salt)	C_26_H_45_NO_6_S	0.73	499.29712	7.004	↑^a^	9.11	↑	0.51
4	Stercobilin	C_33_H_46_N_4_O_6_	1.37	594.34255	6.96	↑^d^	1.99	↓	−0.70
5	sn-3-O-(Geranylgeranyl)glycerol1-phosphate	C_5_H_14_NO_6_P	0.53	215.05534	0.915	↑^d^	2.43	↓	−2.63
6	Vitamin B2	C_17_H_20_N_4_O_6_	−0.01	376.13828	5.503	↑^a^	3.96	↓	−0.48
7	Pseudouridine	C_9_H_12_N_2_O_6_	0.26	244.0696	0.959	↑^a^	1.95	↑	3.02
8	Prostaglandin E2	C_20_H_32_O_5_	0.44	352.22513	7.929	↑^a^	5.50	↑	0.32
9	Prostaglandin D2	C_20_H_32_O_5_	−6.59	352.22265	8.092	↓^a^	−7.23	↑	6.21
10	N-Acetylvanilalanine	C_12_H_15_NO_5_	−0.69	253.09485	5.004	↑^a^		↓	
11	L-Glutamine	C_5_H_10_N_2_O_3_	−7.72	146.06801	1.031	↑^a^	3.60	↓	−1.00
12	Hypoxanthine	C_5_H_4_N_4_O	−9.37	136.03738	0.973	↑^d^	6.54	↓	−1.16
13	Glycerophospho-N-palmitoyl ethanolamine	C_21_H_44_NO_7_P	−0.17	453.28546	11.984	↑^a^	4.77	↓	−2.73
14	Docosahexaenoyl glycine	C_24_H_35_NO_3_	0.26	385.26179	15.961	↑^a^	2.68	↓	−2.52
15	DL-Malic acid	C_4_H_6_O_5_	−9.43	134.02033	0.971	↑^a^	7.90	↓	−0.01
16	Deoxycholic acid	C_24_H_40_O_4_	−7.04	392.2899	8.793	↓^a^	−5.74	↑	1.26
17	D-Glucose 6-phosphate	C_6_H_13_O_9_P	0.5	260.02985	1.023	↑^a^	3.66	↓^c^	−2.31
18	D-Glucosamine 6-phosphate	C_6_H_14_NO_8_P	1.11	259.04555	0.881	↑^a^	8.32	↓^b^	−1.83
19	Citric acid	C_6_H_8_O_7_	−4.84	192.02607	1.254	↑^a^	3.58	↓	−0.19
20	Citraconic acid	C_5_H_6_O_4_	−9.67	130.02535	1.254	↑^a^	1.19	↓	−0.59
21	6-Ketoprostaglandin F1α	C_20_H_34_O_6_	−0.31	370.23542	7.372	↑^a^	1.88	↓	−0.89
22	4-vinylphenol sulfate	C_8_H_8_O_4_S	−2.38	200.01385	6.422	↑^a^	2.94	↓	−0.96
23	4-ethylphenylsulfonic acid	C_8_H_10_O_4_S	−1.8	202.02962	6.642	↑^a^	4.37	↓	−0.85
24	4-Dodecylbenzenesulfonic acid	C_18_H_30_O_3_S	−6.5	326.18944	14.055	↓^a^	−8.98	↓	−0.55
25	2-methoxyacetaminophen sulfate	C_9_H_11_NO_6_S	−1.03	261.03044	1.323	↑^d^	2.37	↓	−1.17
26	2-Dehydro-D-gluconate	C_6_H_10_O_7_	−3.31	194.04201	0.938	↑^a^	5.00	↓	−0.68
27	2′-Deoxyinosine	C_10_H_12_N_4_O_4_	0.56	252.086	1.374	↑^a^	5.44	↓	−1.36
28	1-tetradecyl-sn-glycero-3-phosphocholine	C_22_H_48_NO_6_P	−0.54	453.32168	15.679	↑^a^	6.20	↓	−1.36
29	(±)9-HpODE	C_18_H_32_O_4_	0.18	312.23012	10.605	↑^a^	3.54	↓	−0.35
30	(2R_3S)-2_3-Dimethylmalate	C_6_H_10_O_5_	−4.47	162.0521	1.416	↑^a^	8.01	↓	−1.12

^d^ *p* < 0.05.

^a^ *p* < 0.05 vs. Control group.

^b^ *p* < 0.05.

^c^ *p* < 0.01 vs. Model group.

### 3.7 Metabolic pathway analysis

**FIGURE 7:**
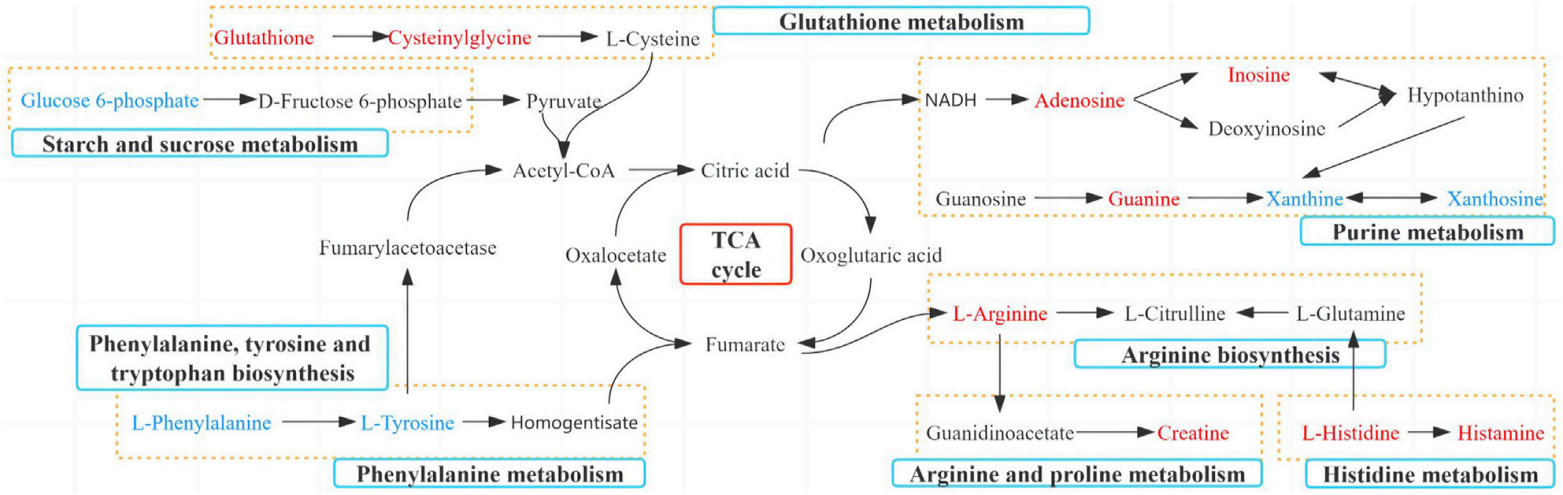
Metabolic pathways significantly regulated by TGP in small intestinal tissue. Red: increased metabolites level; blue: decreased metabolites level; blue box: metabolic pathway of metabolites.

The screened potential biomarkers were imported into MetaboAnalyst 5.0 for pathway analysis, and a total of 18 were enriched, as shown in Figure 6B. The top eight pathways that impacted the bubble map were phenylalanine, tyrosine and tryptophan biosynthesis, phenylalanine metabolism, histidine metabolism, starch and sucrose metabolism, arginine and proline metabolism, arginine biosynthesis, glutathione metabolism, and purine metabolism. The metabolic pathway map is shown in Figure 7.

### 3.8 Network pharmacology analysis

**FIGURE 8:**
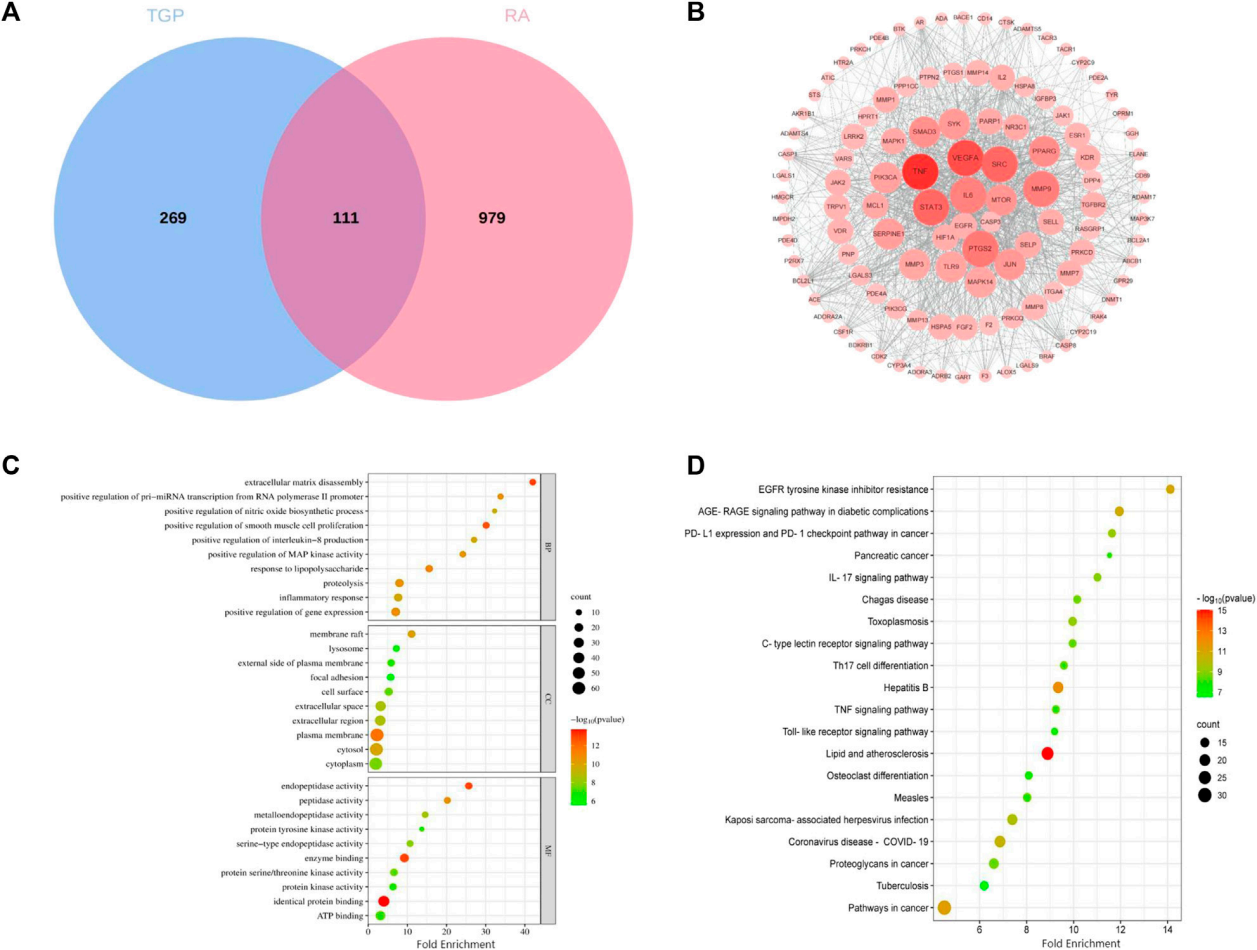
Network pharmacology analysis of TGP in the treatment of RA. **(A)** Components and disease intersection target venn diagram. **(B)** PPI interaction network responsible for 111 potential targets of TGP against RA. Each node represents a protein, and the connections between nodes represent the interactions between proteins. **(C)** GO biological process analysis of potential targets. **(D)** The KEGG enrichment analysis of potential targets. The abscissa indicates the number of targets in the signaling pathway, the size of the bubble indicates the number of genes in the pathway; the ordinate indicates the signaling pathway; the color of the bubble indicates the *p*-value, and red indicates a larger *p*-value.

For the 10 monoterpenes and their glycosides components in TGP, 380 target genes were obtained from the SwissTargetPrediction and Similarity Ensemble Approach databases. 1,090 related genes of RA were obtained from Therapeutic Target Database, DisGeNET and Drugbank databases. After intersection of 380 targets with 1,090 genes, 111 targets were identified as potential targets of TGP for RA (Figure 8A). The intersection targets were entered into the String database to build a PPI network diagram, as shown in Figure 8B. There were 111 nodes and 1,230 edges in PPI network graph, the average node degree of freedom were 22.6, and the average local clustering coefficient was 0.599. According to the MCC algorithm, VEGFA, CASP3, JUN, HIF1A, STAT3, EGFR, TNF, IL6, SRC, MTOR are the top 10 genes.

Then we used the DAVID database to perform GO and KEGG pathway enrichment analysis on the above 111 targets, and enrichment results are sorted by *p*-value. GO analysis mainly includes three parts: biological process (BP), cellular component (CC) and molecular function (MF). As shown in Figure 8C, The top ten results with the smallest *p*-values are visualized. BP mainly includes extracellular matrix disassembly, positive regulation of smooth muscle cell proliferation and response to lipopolysaccharide, etc.,; CC mainly includes eplasma membrane, membrane raft and cytosol, etc.,; MF mainly includes identical protein binding, enzyme binding and endopeptidase activity, *etc.* The most important pathways in KEGG enrichment pathways are lipid and atherosclerosis, hepatitis B pathways in cancer, EGFR tyrosine kinase inhibitor resistance, AGE-RAGE signaling pathway in diabetic complications, coronavirus disease-COVID-19, kaposi sarcoma-associated herpesvirus infection, PD-L1 expression and PD-1 checkpoint pathway in cancer, toxoplasmosis, etc (Figure 8D).

### 3.9 Joint analysis of network pharmacology and metabonomics

**FIGURE 9:**
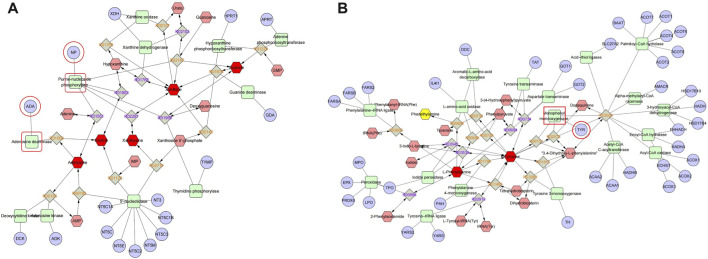
Compound-reaction-enzyme-gene networks for purine metabolism **(A)** and tyrosine metabolism **(B)**. Red hexagons, gray diamonds, green rounded rectangles, and purple circles represent active compounds, reactions, proteins, and genes, respectively.

To obtain the associated genes of differential metabolites, 32 TGP-regulated differential metabolites were imported into Metscape to obtain a compound-reaction-enzyme-gene network. 163 potential target genes were found in the compound-reaction-enzyme-gene network. By intersecting the above 163 targets with 111 targets in the PPI network, 3 key targets were identified, including adenosine deaminase (ADA), purine nucleoside phosphorylase (PNP) and tyrosinase enzyme (TYR). As shown in Figures 9A,B, ADA and PNP are involved in purine metabolism pathway while TYR is involved in tyrosine metabolism.

### 3.10 Molecular docking and verification of common targets

ADA, PNP and TYR were identified as common targets based on metabonomics and network pharmacology analysis. Immunofluorescence and RT-qPCR were used to analyze the expression of ADA, PNP and TYR in rat small intestine. Since the expression of TYR gene was too low in the small intestine, it is difficult to detect it in RT-qPCR experiments, so only the expression of ADA and PNP was analyzed in this study. According to the results of network pharmacology, we carried out molecular docking of ADA and PNP with four corresponding TGP components (paeoniflorin, paeoniflorin, oxidized paeoniflorin, and 8-debenzoylpaeoniflorin). The lower the binding energy between the ligand and the receptor, the more stable the conformation is. Generally, the binding energy < −5 kcal/mol is used as the standard (Li et al., 2022). As shown in Table 3, the binding energies of the above components to ADA and PNP were all less than −5 kcal/mol, indicating a strong affinity between the receptor and the ligand. Supplementary Figure S5 shows the hydrogen bonds formed between each component and key targets and the binding sites of amino acid residues.

**TABLE 3:** Binding energy between TGP and key targets.

Target	Compound	Number of hydrogen bonds	Affinity (kcal/mol)	Amino acid residue
ADA	Paeoniflorin	1	−6.1	LYS-170
	8-Debenzoylpaeoniflorin	2	−6.0	ALA-279 LEU-243
PNP	Paeoniflorin	2	−6.1	GLN-269 ARG-185
	Albiflorin	2	−6.3	PRO-54 AGR-58
	Oxidized paeoniflorin	2	−6.5	GLN-82
	8-Debenzoylpaeoniflorin	3	−6.4	AGR-158 ASN145 PHE141

**FIGURE 10:**
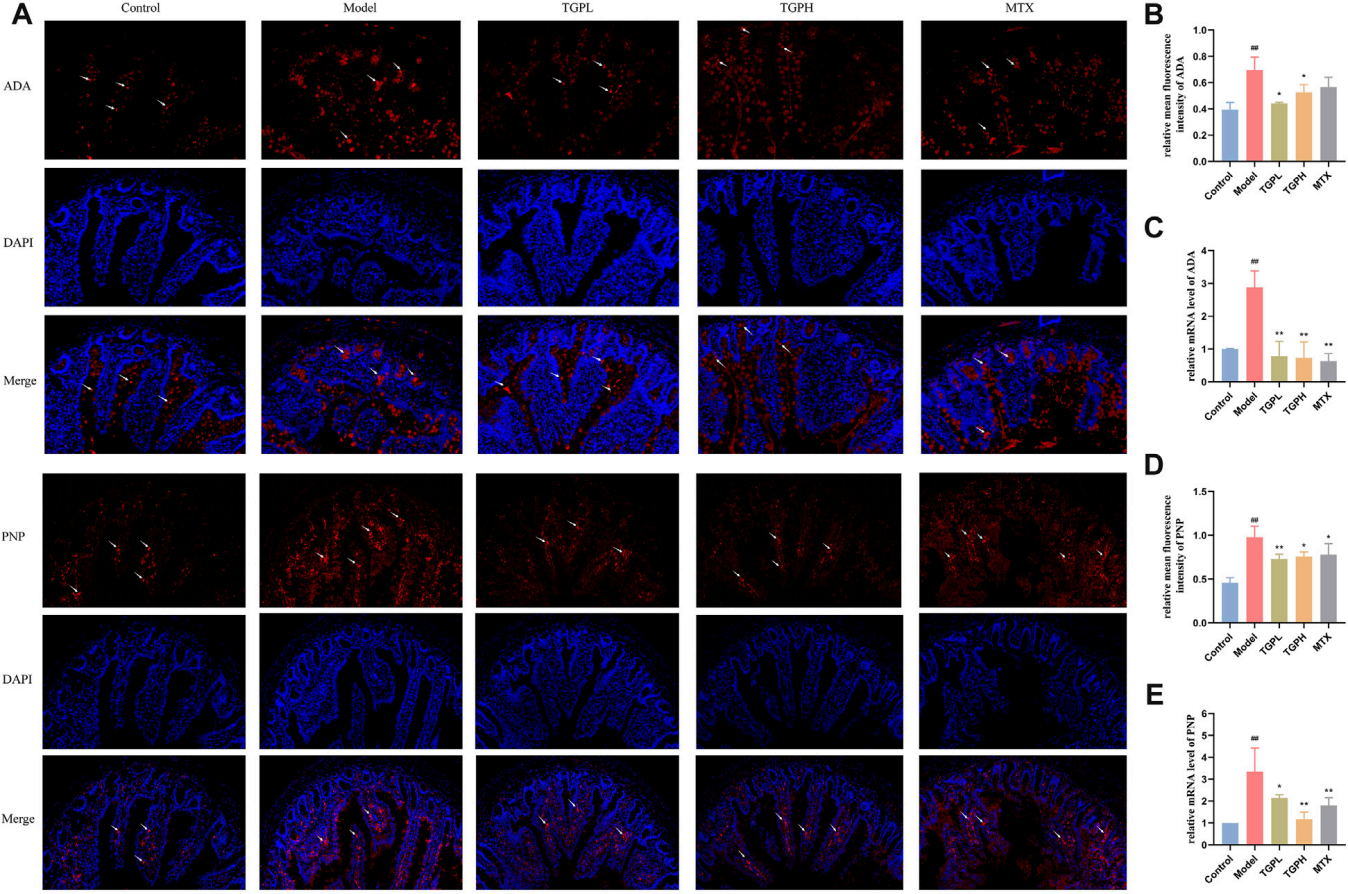
Effect of TGP on the levels of ADA and PNP in small intestine of rats. **(A)** Immunofluorescent staining images of ADA and PNP after TGP administration. Relative mean immunofluorescence intensities of ADA **(B)** and PNP **(D)** (n = 4). mRNA expression levels of ADA **(C)** and PNP **(E)** (n = 4). ^#^ *p* < 0.05, ^##^ *p* < 0.01 vs. control group; ^*^ *p* < 0.05, ^**^ *p* < 0.01 vs. model group.

The expression of ADA and PNP in rat small intestine was further detected by immunofluorescence and RT-qPCR. As shown in Figures 10A, B, D, the expressions of ADA and PNP in the model group were significantly increased compared with the control group. The contents of ADA and PNP were significantly reduced after TGP administration. There was no significant difference in the expression levels of ADA and PNP between the positive drug and the model group. In addition, we analyzed the expression of the above genes by RT-qPCR and obtained similar results (Figures 10C, E).

## 4 Discussion

The intestinal mucosa in the organism is the first line of defense in contact with many external antigens. The intestinal epithelial immune system mainly functions through gut-associated lymphoid tissue, including PPs, LPLs and IELs (Jørgensen et al., 2021). Mature T cells can be divided into CD4^+^ T cells and CD8^+^ T cells according to different phenotypes. CD4^+^ T cells assist B cells to differentiate and produce antibodies, while CD8^+^ T cells can kill and inhibit target cells (Wu et al., 2022). CD4^+^/CD8^+^can preliminarily assess the immune status (Revillard and Berard-Bonnefoy, 1993). In our previous studies, we demonstrated that TGP intervention could regulates intestinal microbial imbalance, Th1/Th2 and Th17/Treg ratio imbalance in peripheral blood and inhibits intestinal cytokines SIgA and IFN-γ in CIA rats (Peng et al., 2019). Previous studies also have shown that paeoniflorin, the main component of TGP, exerts anti-arthritic effects mainly by regulating the proportion of Th1 and Th17 in mesenteric lymph nodes and PPs of CIA rats, rather than in the spleen (Fei et al., 2019). Nevertheless, TGP is a drug with slow onset, long treatment period and complex intestinal immune action. At present, there is still a lack of research on the dynamic changes of different intestinal mucosal immune sites in different TGP administration cycles. To this end, we confirmed that TGP indeed has the potential to reduce the lymphocyte immune response in the small intestine to generate immune tolerance, and the effects of TGP in different parts of intestinal mucosal immunity. During the development of RA, INF-γ can promote the development of Th1 cells, inhibit the activation and proliferation of Th2 cells, increase the level of osteoclast factors and lead to bone destruction (Tang et al., 2018). Therefore, TGP inhibits IFN-γ by regulating the balance of intestinal T lymphocytes, which may be the possible mechanism for it to regulate the Th1/Th2 ratio of peripheral blood in CIA rats to improve RA. However, the regulation of TGP is not synchronized at different sites of lymphocyte synthesis. IELs and LPLs, as the lymphocyte population closer to the intestinal lumen antigen, showed the earliest difference between the model group and the control group, and the PPs lymphocytes changed later. It was also monitored that the TGP action time for different T cells was not consistent. These results may be the IELs and LPLs were more likely to be exposed to TGP after oral administration, so the overall effect of TGP on IELs and LPLs was earlier than PPs lymphocytes. The above results further proved the hypothesis that TGP acts directly on the intestine.

The intestinal epithelial barrier has the function of preventing bacteria and other pathogens from entering the systemic circulation from intestinal tissues. Clinical studies have shown that many patients with autoimmune diseases such as RA, multiple sclerosis, and ankylosing spondylitis are accompanied by increased intestinal permeability (Ciccia et al., 2017; Buscarinu et al., 2018). The destruction of intestinal barrier breaks the balance between non autoantigens and intestinal immunity, and immune cells migrate from the intestine to the joints, thus inducing RA (Tajik et al., 2020). Increasing the permeability of intestinal epithelial barrier by targeting intestinal barrier function may be a new target for treating RA (Matei et al., 2021). Current studies have shown that TGP can protect the intestinal epithelial barrier of animals with inflammatory bowel disease. Its mechanism of action may be related to the inhibition of the Lyn/Snail signaling pathway (Cao et al., 2021), PI3K-AKT-mTOR (Ma et al., 2023) signaling pathway. In this study, we observed varying degrees impairment of intestinal epithelial barrier in CIA rats, manifested in shortened villi length and increased crypt depth. According to the pathological sections of rat small intestine, TGP administration can repair damaged small intestinal epithelial villi and increase the ratio of V/C. TJ proteins is the main form of intercellular junctions in the intestinal epithelium and an important structure constituting the epithelial mechanical barrier. ZO-1 and occludin proteins are two important proteins constituting tight junction proteins. Studies have shown that tight junction integrity is regulated by cytokines TNF-α and IFN-γ, and the IFN-γ can inhibit the expression of ZO and occludin to increase intestinal permeability (Scharl et al., 2009). TGP can increase the protein and gene expression levels of ZO-1 and occludin in the small intestine, which may be mediated by the inhibition of IFN-γ expression by TGP (Peng et al., 2019). In conclusion, impaired epithelial barrier in the small intestine of CIA rats can be ameliorated by TGP. However, MTX has been reported to have general gastrointestinal toxicity, it consequently had no apparent effect on the small intestinal epithelial barrier in CIA rats (Beutheu et al., 2012).

Most of RA metabolomics focused on the analysis of biological fluids such as plasma, urine, *etc.* In this study, we explored the regulatory effect of TGP on the metabolic profile of the small intestine of CIA rats. Based on the metabolomics analysis we identified a total of 32 differential metabolites that were significantly reversed by TGP, and discovered 8 key metabolic pathways, including phenylalanine, tyrosine and tryptophan biosynthesis, phenylalanine metabolism, histidine metabolism, starch and sucrose metabolism, arginine and proline metabolism, arginine biosynthesis, glutathione metabolism, and purine metabolism. Some metabolic pathways have been confirmed to be directly or indirectly involved in inflammation and immune response in the pathogenesis of RA (Xu et al., 2022). Catecholamines are a class of neurohormones including dopamine, norepinephrine, and epinephrine, which can be produced from phenylalanine and tyrosine by the action of tyrosine hydroxylase. Previous studies have shown that lymphocytes themselves could synthesize and release catecholamines, and selectively regulate lymphoid subsets of T cells through autocrine/paracrine feedback fashion (Flierl et al., 2008). TGP could downregulate L-tyrosine and L-phenylalanine in the small intestine of CIA rats, suggesting that TGP may regulate intestinal immunity by regulating tyrosine metabolism and then decreasing the release of catecholamines from T lymphocytes. Arginine metabolism and synthesis is another metabolic pathway related to the anti-RA effect of TGP. In our study, TGP can increase the level of L-arginine in the small intestine of CIA rats and interfere with the synthesis and metabolism of arginine. Existing studies have shown that, when the immune response is activated, M1 macrophages actively take up extracellular arginine and produce NO through nitric oxide synthase 2 (NOS2) (Qualls et al., 2012). NO can regulate mitochondrial membrane potential in human T cells and mitochondrial production in human lymphocytes, thereby breaking the balance of Th1/Th2 and Th17/Treg cells (Talaat et al., 2015). Our previous studies have confirmed that TGP can downregulate the levels of Th1 cells and Th17 cells and upregulate the levels of Th2 cells and Treg cells in peripheral blood (Peng et al., 2019). This effect of maintaining the balance of Th1/Th2 and Th17/Treg cells may be related to the increase of L-arginine level to resist the uptake of M1 cells and produce NO. Moreover, the metabolites of arginine play an important role in maintaining the intestinal barrier and promoting the repair of the gastrointestinal mucosa (Andrade et al., 2015). At the same time, some studies have shown that a variety of inflammatory lesions and immune disorders, including RA, are related to changes in the intracellular level of glutathione (Kidd, 2003; Chávez and Tse, 2021). Glutathione have been confirmed by research that it can regulate the activity of centriocytes and dendritic cells, and glutathione depletion can inhibit lymphocyte CD8^+^ cells and activate CD4^+^ T lymphocytes, leading to inflammatory/immune-mediated diseases (Gmünder and Dröge, 1991). The level of reduced glutathione in intestinal tissue increased significantly and the ratio of CD4^+^/CD8^+^ in different parts of intestinal mucosal immunity decreased significantly after 8 weeks of TGP administration in this study, suggesting that TGP may regulate lymphocytes in intestinal mucosal immunity and epithelial barrier through glutathione metabolism, thereby exerting an anti-RA effect.

Network pharmacology is an emerging discipline integrating systems biology, pharmacology and biochemistry. It is based on the “molecule (drug)-target-pathway-disease” network to study the mechanism of drug, and is considered to be an important tool for modern pharmacology research of traditional Chinese medicine (Zhang R. et al., 2019). Nevertheless, network pharmacology is still limited by the completeness and reliability of public databases, and the screened pathways and targets still need further experimental verification (Zhou et al., 2020). In recent years, many studies have comprehensively analyzed metabolomics and network pharmacology, and the two methods refer to each other, which has brought great help to the study of drug mechanism (Wei et al., 2020; Qu et al., 2021). In this study, in order to more accurately explore the mechanism which TGP acts on intestinal immunity and barriers, the integrated analysis combining metabolomics and network pharmacology was used to construct a compound-reaction-enzyme-gene network, and the two common targets of ADA and PNP were finally determined. Notably, ADA and PNP are essential targets involved in purine metabolic pathways. Many studies have found that there are purine metabolism abnormalities in RA patients and animal models, which are manifested by the increase of the end product uric acid level (Lee et al., 2016; Zhan et al., 2020). Our study showed that TGP could significantly reverse the abnormalities in the levels of major components of purine metabolism in the small intestine of CIA rats, including adenosine, xanthine, hypoxanthine, inosine, and guanine. Studies have shown that binding of adenosine to A2A receptors can inhibit T cell receptors and lead to inactivation of T cells, thereby reducing inflammation levels and tissue damage (Ochaion et al., 2008). It also activates cAMP response elements, which enable CD4^+^ and CD8^+^ T cells from myeloid cells to produce TGF-β and IL-10 (Cronstein and Sitkovsky, 2017). In addition, hyperuricemia model mice caused by abnormal purine metabolism can also cause intestinal epithelial damage and reduce the expression of ZO-1 and occludin (Guo et al., 2019). Coincidentally, our results also found the potential of TGP to increase the levels of adenosine and its downstream metabolite inosine in CIA rats. ADA can deaminate adenosine and converts it to inosine, which can then be derivatized by the enzyme PNP to convert it to hypoxanthine. Therefore, the contents of ADA and PNP in the small intestine were further analyzed. The expressions of ADA and PNP in the small intestine of CIA rats were significantly reduced after TGP treatment. ADA plays an important role in the maintenance and maturation of the immune system, by regulating the catabolism of adenosine (Whitmore and Gaspar, 2016). A large number of clinical studies shown that the level of ADA in the blood of RA patients was significantly increased (Gao et al., 2021). Recent studies shown that ADA could lead to metabolic remodeling in RA, further mediating the death of chondrocytes, the proliferation of synoviocytes, and the differentiation of macrophages into osteoclasts (Bhagavatham et al., 2021). PNP is another enzyme that mediates purine metabolism through the purine salvage pathway. Abnormal expression of PNP will lead to blockage of the degradation of purine nucleosides inosine and guanosine, causing accumulation of metabolites in mitochondria, blockage of DNA synthesis and abnormal cell function, and participate in the occurrence of inflammation and autoimmune diseases (Torun et al., 2022). In summary, ADA and PNP through the purine metabolic pathway may be the possible targets of TGP to regulate intestinal immunity and protect the epithelial barrier.

## 5 Conclusion

In conclusion, our study showed that long-term administration of TGP can effectively suppress the intestinal mucosal immune response and improve the intestinal epithelial barrier in CIA rats. In particular, through dynamic monitoring of multiple time points and multiple sites, the role and regularity of TGP in maintaining intestinal mucosal immune balance were further elucidated. Furthermore, by integrating metabolomics and network pharmacology, 32 biomarkers were obtained, and a key metabolic pathway (purine metabolism) and two key targets (ADA and PNP) were identified. This study provided a new insight for clarifying the potential target organ and immune regulation mechanism of TGP in the treatment of RA.

## Data Availability

The datasets presented in this study can be found in online repositories. The names of the repository/repositories and accession number(s) can be found in the article/Supplementary Material.
