# Supplementary material for: Prolonged administration of total glucosides of paeony improves intestinal immune imbalance and epithelial barrier damage in collagen-induced arthritis rats based on metabolomics-network pharmacology integrated analysis
Source: Front Pharmacol. 2023 Nov 13;14:1187797. doi: 10.3389/fphar.2023.1187797 (PMC10679728; doi:10.3389/fphar.2023.1187797)
Supplement: Supplementary file 1 [file DataSheet1.pdf]

Supplementary Material

Supplementary Table S1. Primer sequence for real-time quantitative PCR analysis (RT-qPCR).

No.	Gene	Primer Sequence
1	ZO-1	Forward: 5’-CATGAGAAGCAGACACCCAC-3’
		Reverse: 5’-TGTTGAATTTGGCAGAACAC-3’
2	Occludin	Forward: 5’-TCCACCTATCACTTCAGA-3’
		Reverse: 5’-TCGTCGAGTTCTGCCAGTAA-3’
3	ADA	Forward: 5’-AGACGCCCGCATTCAACAAG-3’
		Reverse: 5’-GGTCGATTCCTCTCTTCTTGCC-3’
4	PNP	Forward: 5’-GGTACTCGCTGTCAAAGGTGA-3’
		Reverse: 5’-TGATGTGGTCACGGATCAGC-3’
5	β-actin	Forward: 5’-AGTACTCTGTGTGGATTGGT-3’
		Reverse: 5’-CAGGAGTACGATGAGTCCGG-3’

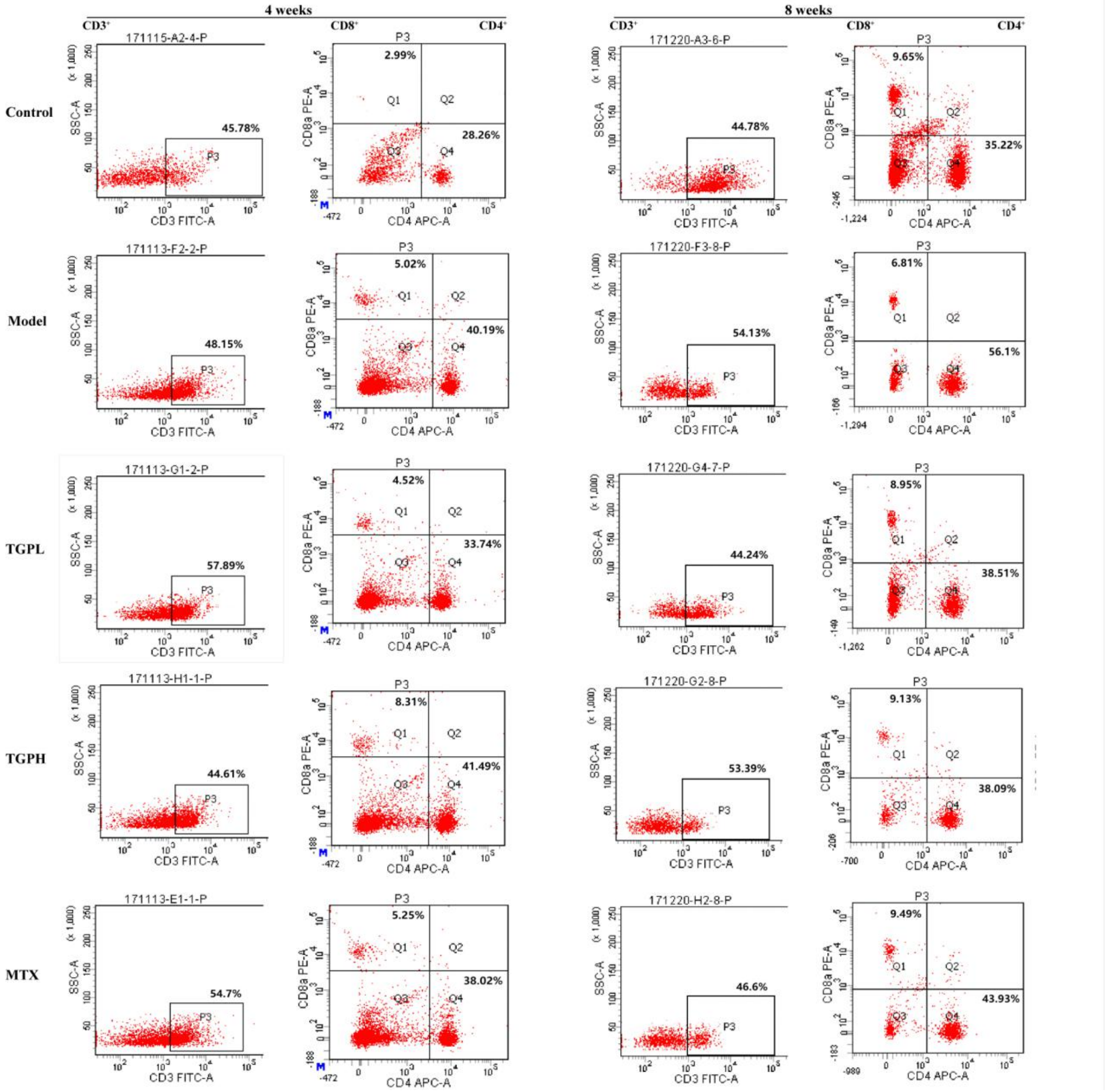

Supplementary Figure S1. Flow cytometry dot plot analyses of CD3+, CD4+, and CD8+ in PPs lymphocytes.

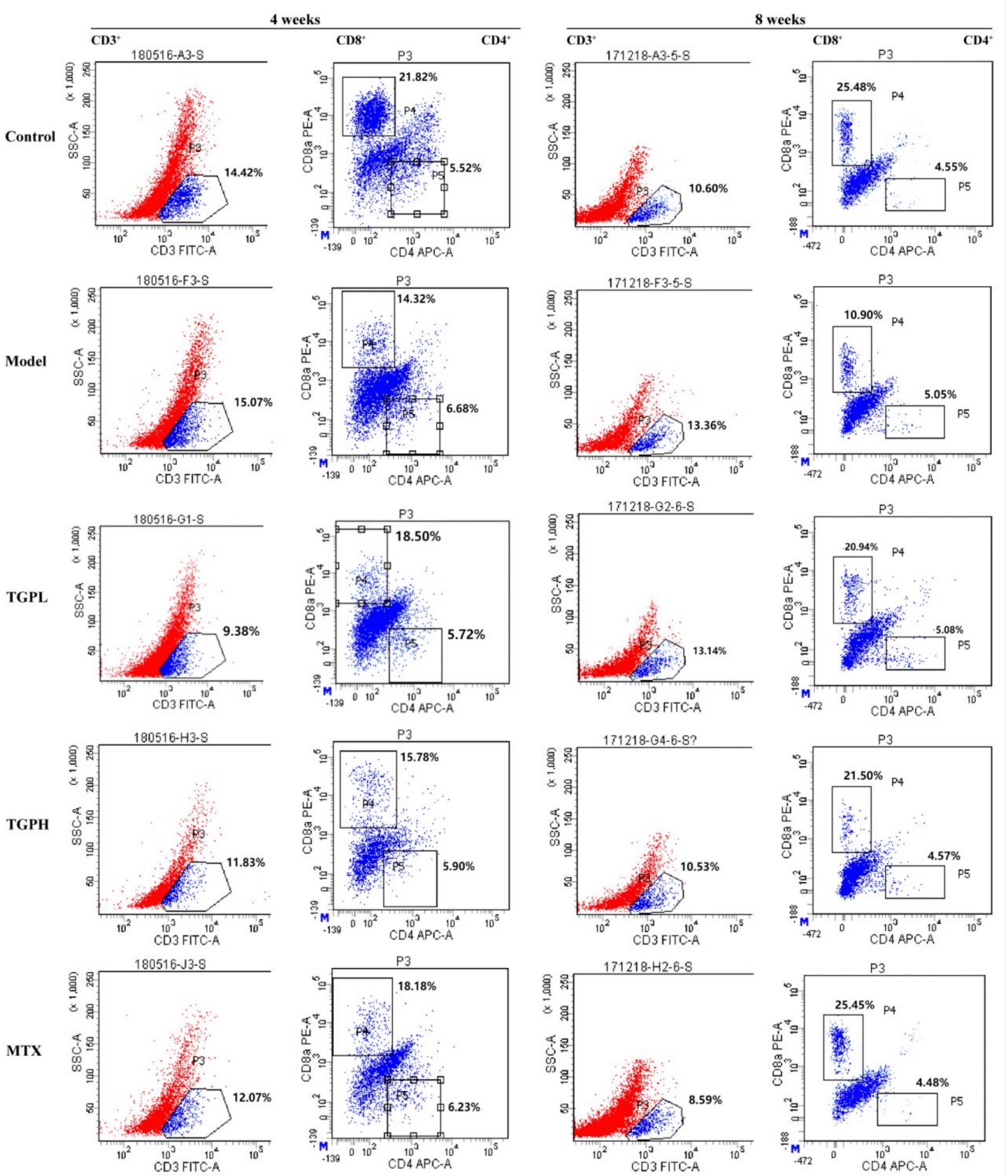

Supplementary Figure S2. Flow cytometry dot plot analyses of $CD3^+$, $CD4^+$, and $CD8^+$ in LELs.

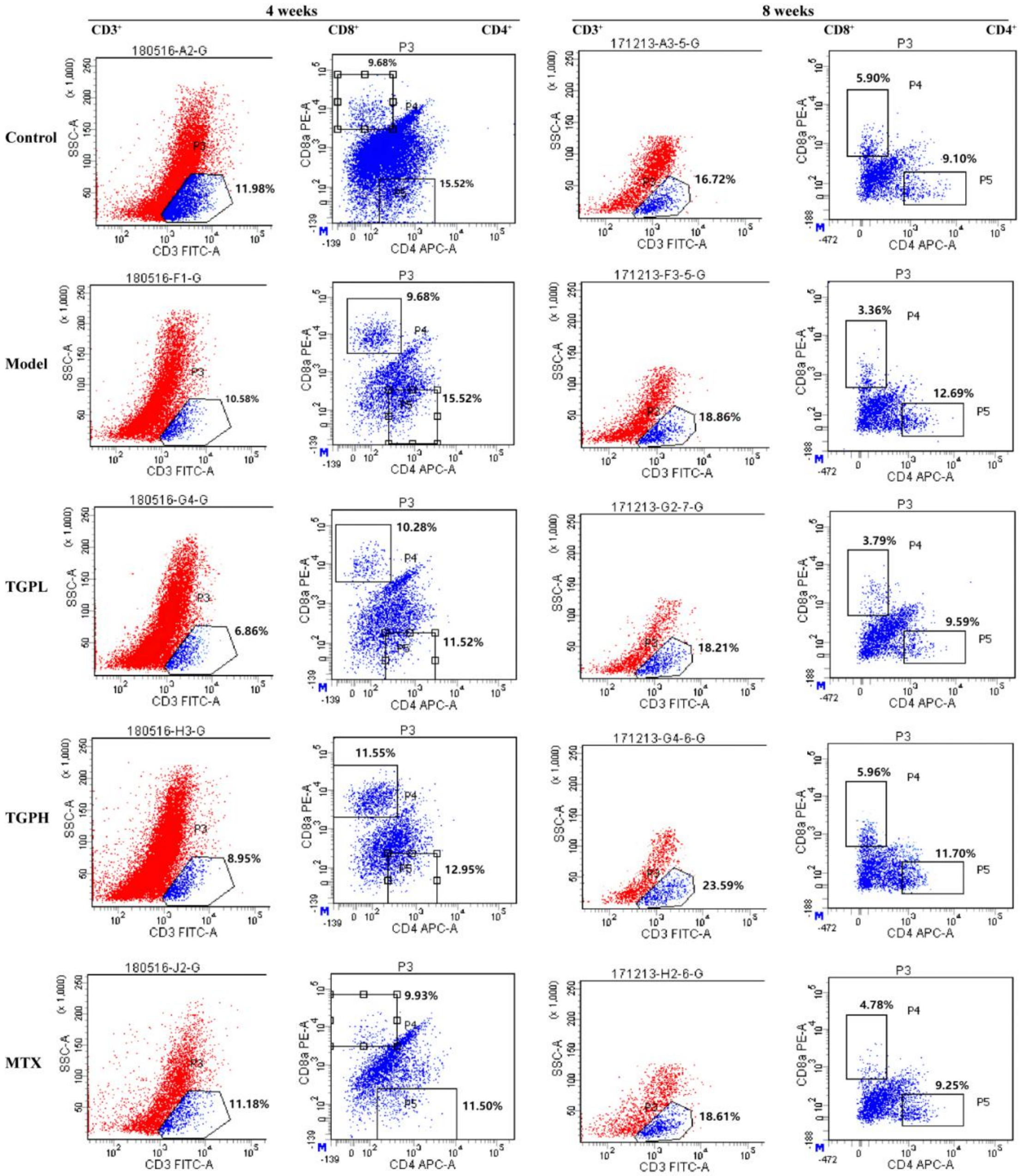

Supplementary Figure S3. Flow cytometry dot plot analyses of $CD3^+$, $CD4^+$, and $CD8^+$ in LPLs.

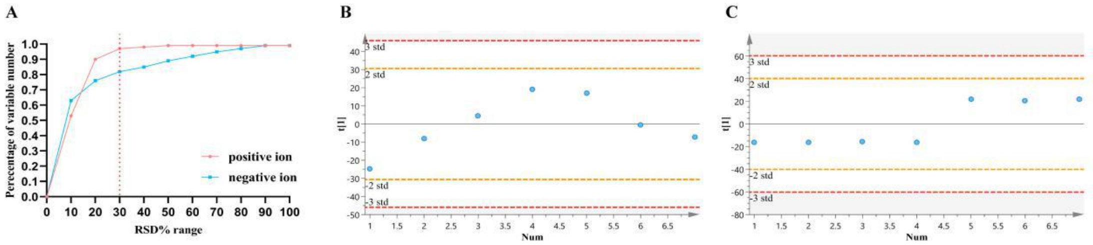

Supplementary Figure S4. LC-MS analysis variation determined by QC samples. **(A)** Distribution of RSD% of accumulative percentage for detected peaks in the pooled QC samples. The first PCA component t[1] of QC samples in positive **(B)** and negative **(C)** ion modes.

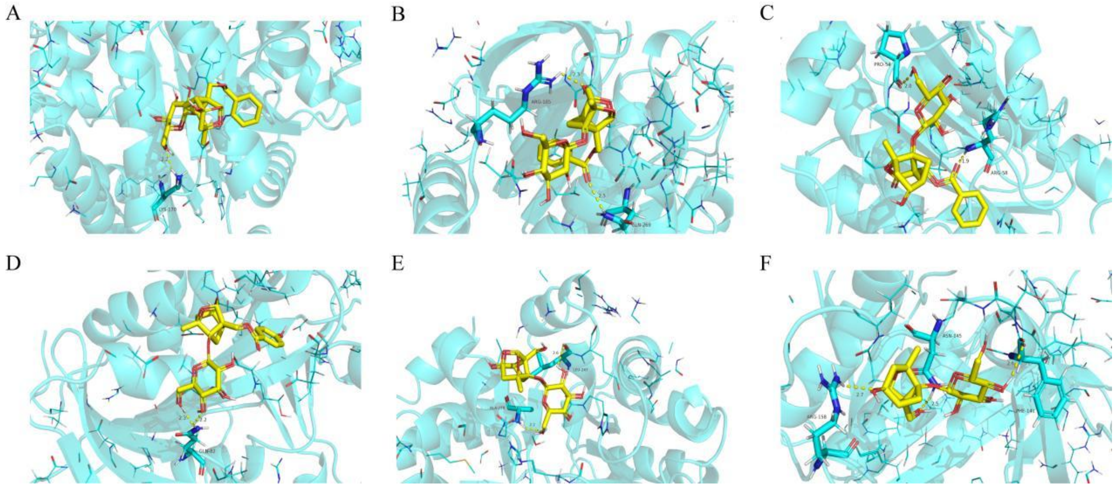

Supplementary Figure S5. Visualization of molecular docking results. **(A)** Paeoniflorin and ADA; **(B)** Paeoniflorin and PNP; **(C)** Albiflorin and PNP; **(D)** Oxidized paeoniflorin and PNP; **(E)** 8-Debenzoylpaeoniflorin and ADA; **(F)** 8-Debenzoylpaeoniflorin and PNP
